# The prevalence of orthostatic intolerance, postural orthostatic tachycardia syndrome and orthostatic hypotension in post-acute sequelae of COVID-19

**DOI:** 10.3389/fcvm.2025.1679252

**Published:** 2026-01-08

**Authors:** Chunliang Wang, Yuzhu Fan, Chang Li, Bing Chang, Juan Wang, Xu Cao, Guiting Liang, Yan Liang, Kan Sun

**Affiliations:** 1 Department of Cardiology, Shijiazhuang Traditional Chinese Medicine Hospital, Shijiazhuang, China; 2 Department of Endocrinology, Shijiazhuang Traditional Chinese Medicine Hospital, Shijiazhuang, China; 3 Department of Pharmacy, Shijiazhuang Traditional Chinese Medicine Hospital, Shijiazhuang, China; 4 Department of Endoscopy Center, Shijiazhuang Traditional Chinese Medicine Hospital, Shijiazhuang, China

**Keywords:** COVID-19, orthostatic hypotension, orthostatic intolerance, posturalorthostatic tachycardia syndrome, prevalence

## Abstract

**Background:**

The COVID-19 pandemic has resulted in over 776 million confirmed cases worldwide, with post-acute sequelae of COVID-19 now recognized as a significant public health concern. Autonomic dysfunction—including orthostatic intolerance (OI), postural orthostatic tachycardia syndrome (POTS), and orthostatic hypotension (OH)—constitutes a major complication of post-acute sequelae of COVID-19. However, reliable epidemiological estimates remain scarce. As a result, we aim to provide the first global prevalence estimates of autonomic dysfunction in post-acute sequelae of COVID-19.

**Methods:**

This systematic review and meta-analysis adhered to PRISMA 2020 guidelines, including 21 observational studies. Random-effects models were utilized to estimate pooled prevalence, and sensitivity and meta-regression analyses were conducted to explore heterogeneity. GRADE assessments evaluated evidence certainty.

**Results:**

The pooled prevalence estimates demonstrated 70.6% for OI, 36.2% for POTS, and 18.6% for OH. Advancing age exhibited a significant negative association with POTS and OH. In contrast, prolonged post-acute sequelae of COVID-19 duration and female sex showed no significant association with the incidence rates of these conditions. Subgroup analyses indicated higher POTS and OH incidence in mild vs. moderate or severe COVID-19 cases. Publication bias was minimal for OH but evident for POTS.

**Conclusion:**

This study provides the first global prevalence estimates of autonomic dysfunction in post-acute sequelae of COVID-19, highlighting its disproportionate burden among younger populations and non-linear temporal trends. The findings advance epidemiological understanding and inform evidence-based public health strategies to address post-COVID complications.

**Systematic Review Registration:**

https://www.crd.york.ac.uk/PROSPERO/display_record.php?RecordID=556546, PROSPERO CRD42024556546.

## 1 Introduction

Since the onset of the COVID-19 pandemic, more than 776 million cases have been reported globally (1). COVID-19 affects not only the respiratory system but also extrapulmonary organs, including the nervous and the cardiovascular system (2–6). A growing body of evidence demonstrates that persistent, relapsing, or novel symptoms and conditions—collectively termed post-acute sequelae of COVID-19 (PASC) or long COVID—persist for more than 12 weeks post-infection (7, 8). Autonomic dysfunction, a common complication of PASC, includes orthostatic intolerance (OI), postural orthostatic tachycardia syndrome (POTS), orthostatic hypotension (OH), and associated symptoms such as chest discomfort, dyspnea, fatigue and disturbances in sleep (9, 10). OI refers to a spectrum of conditions characterized by symptoms provoked by upright posture, including OH and POTS. The diagnosis of POTS necessitates the exclusion of OH, indicating that patients may present with either OH or POTS, but not both simultaneously (11). OI, POTS, and OH collectively impose substantial burdens on health-related quality of life. These conditions are linked to elevated risks of falls (12), cardiovascular events (13), cognitive decline (14, 15), and multisystemic morbidity (16). However, reliable estimates of the pooled prevalence of OI, POTS, and OH remain lacking, due to limited systematic quantitative syntheses. Although publication volume in this field has grown recently, reported prevalence estimates exhibit marked heterogeneity. Jamal et al. performed a prospective cohort study assessing cardiac autonomic function in PASC patients, reporting prevalences of 62.5% for OI, 16.7% for POTS, and 4.1% for OH (17). In a cross-sectional study, Monaghan et al. evaluated autonomic function in PASC patients, identifying prevalences of 66% for OI, 8.2% for POTS, and 32.9% for OH (18). The authors acknowledged significant heterogeneity and emphasized the need to investigate sources of variability, which likely stem from differences in acute-phase COVID-19 severity, sex-based disparities, inconsistent diagnostic criteria, and heterogeneous PASC durations. However, neither meta-analytical syntheses nor covariate-adjusted meta-regression approaches have been undertaken to systematically explore these critical dimensions.

This systematic review aims to establish contemporary population-based prevalence estimates for OI, POTS, and OH in PASC patients. Additionally, we systematically elucidate heterogeneity across prior studies via stratified meta-analyses and meta-regression modeling.

## 2 Methods

This systematic review was conducted according to the PRISMA 2020 (Preferred Reporting Items for Systematic Reviews and Meta-Analyses) statement (19). The protocol for this article was registered in the International Prospective Register of Systematic Reviews (PROSPERO) under the registration number CRD42024556546.

### 2.1 Search strategy

The PECOS (Population, Exposure, Comparison, Outcomes, Study Design) model was employed to frame the clinical question and develop the search strategy (Supplementary Table S1). Systematic electronic searches were conducted across multiple databases, including PubMed, Embase, and the Cochrane Central Register of Controlled Trials (CENTRAL), for articles published from the databases inception through February 1, 2025. The search strategy utilized a combination of free-text words and Medical Subject Heading terms, adapted to the specific requirements of each database. The reference lists of systematic reviews and shortlisted studies were manually screened to identify any additional citations that may have been overlooked. This search was supplemented with a search of gray literature using Bing, OPENGREY.EU, and ProQuest Dissertations & Theses. No language restrictions were imposed on the search results. These searches were performed independently by two co-authors of this article, who screened the aforementioned electronic databases.

### 2.2 Inclusion criteria

Participants: Individuals were selected among patients with established PASC. PASC is defined as a chronic condition associated with SARS-CoV-2 infection, persisting for ≥3 months as a continuous, relapsing-remitting, or progressive disease state affecting ≥1 organ system (20). Acute-phase COVID-19 severity was classified according to the WHO Clinical Management of COVID-19: Living Guideline (21). Mild COVID-19 was defined as symptomatic patients meeting the case definition of COVID-19 but demonstrating no evidence of viral pneumonia or hypoxia. Moderate COVID-19 applies to adolescents or adults presenting with clinical symptoms of pneumonia (e.g., fever, cough, dyspnea, tachypnea) without signs of severe pneumonia, such as an oxygen saturation (SpO2) ≥90% on room air. Severe COVID-19 is defined as adolescents or adults presenting with clinical symptoms of pneumonia (fever, cough, dyspnea) accompanied by ≥1 of the following: respiratory rate >30 breaths/minute, severe respiratory distress, or SpO2 < 90% on room air.

Exposure: Diagnosis of OI, POTS, or OH. OI is characterized by an inability to tolerate upright posture due to signs and symptoms alleviated by recumbency (22). POTS is diagnosed when a sustained heart rate increase ≥30 bpm occurs in adults (aged >19 years) or ≥40 bpm in children/adolescents (aged ≤19 years) upon transitioning from supine to upright posture within 10 min of standing, with no evidence of classic OH (23). Additionally, studies that detailed the use of objective tests, such as the head-up tilt or active standing test, for assessment and reported POTS diagnoses confirmed by these tests were also included. OH is defined as a sustained reduction in systolic blood pressure (SBP) ≥20 mm Hg or diastolic blood pressure (DBP) ≥10 mm Hg within 3 min of active standing or during a head-up tilt (HUT) test at ≥60 °. Furthermore, based on this criterion of timing, OH is categorized into three subtypes: classic (within 3 min), initial (within 15 s), and delayed (after 3 min) (24)..

Comparator/Control: Not applicable.

Outcomes: The prevalence of OI, POTS, or OH.

Study design: Observational studies were included. However, existing systematic reviews and meta-analyses were excluded. These articles were screened to identify relevant individual studies.

### 2.3 Exclusion criteria

Individuals with acute COVID-19 (symptoms lasting <12 weeks) were excluded. Additionally, those with a pre-existing diagnosis of OI, POTS, or OH prior to SARS-CoV-2 infection were excluded. Non-primary research articles, including book chapters, reviews, and opinion pieces, were excluded *a priori*.

### 2.4 Study selection and data extraction

**Figure 1:**
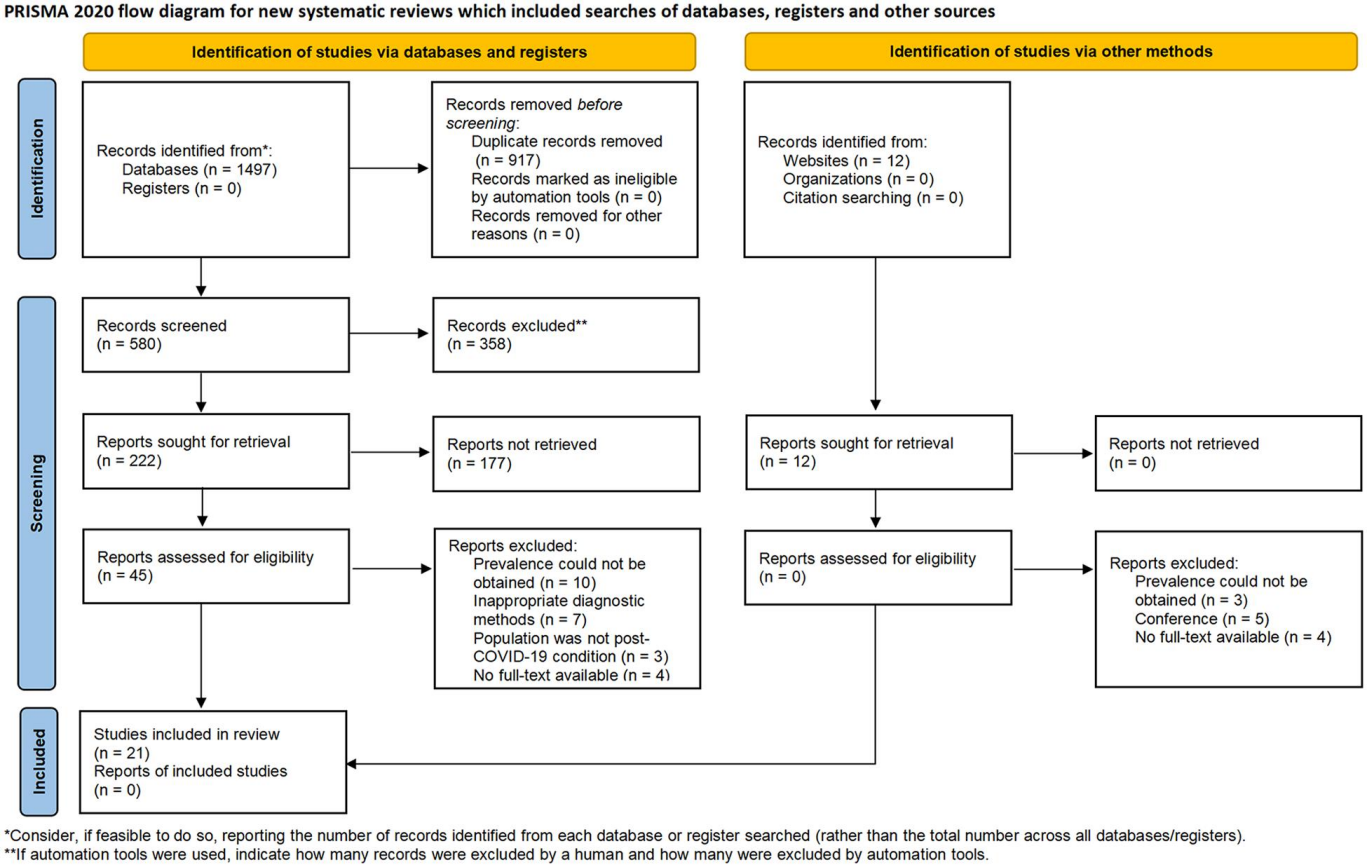
Flow chart of the study selection process.

Two reviewers independently screened all titles and abstracts against the predefined eligibility criteria. Studies deemed potentially relevant underwent full-text review by the same two reviewers to determine final eligibility. When discrepancies in screening decisions occurred, a third reviewer was consulted to adjudicate. Subsequently, two additional reviewers independently extracted data from eligible studies. The extracted data included the following variables: geographic location, study design, sample size, demographic characteristics (age and sex), assessment methodology, prevalence estimates, acute-phase COVID-19 severity classification, and post-COVID-19 follow-up duration. The study selection process is delineated in the PRISMA flow diagram, with exact numbers of included studies presented in Figure 1.

### 2.5 Assessment of risk of bias

According to the Cochrane Collaboration's tool (25), the risk of bias for all the studies included in this review was independently evaluated by two authors using the JBI Critical Appraisal Checklist for studies reporting prevalence data (26). Any disagreements in assessment were resolved through consensus or by a third reviewer. The risk of bias assessment was stratified into nine predefined domains, each categorized using standardized criteria: “yes”, “no”, “unclear”, or “not applicable”. The methodological quality assessment is summarized by domain in Supplementary Table S2.

### 2.6 Statistical analysis

All statistical analyses were performed using R software (version 4.3.2; R Foundation for Statistical Computing) with the meta and metafor packages. Given the anticipated heterogeneity across geographic regions, healthcare settings, and COVID-19 severity classifications, a random-effects model was implemented to estimate pooled prevalences of OI, POTS, and OH. The Generalized Linear Mixed Model (GLMM) framework was adopted as a robust alternative to the Freeman-Tukey double arcsine transformation, per contemporary recommendations for single-proportion meta-analyses (27). Model parameters were estimated via maximum likelihood with Q-profile methods for *τ* (tau) derivation, consistent with Veroniki's methodology. Hartung-Knapp adjustment was consistently applied to optimize confidence interval estimation (28). Between-study heterogeneity was quantified via Cochran's *Q* test and I^2^ statistics. when the results exhibit significant heterogeneity, sensitivity analyses, subgroup analyses, and meta-regression analyses are employed to investigate the sources of heterogeneity. Sensitivity analyses were rigorously implemented via sequential leave-one-out elimination using restricted maximum likelihood estimation. Prespecified subgroup analyses addressed clinical confounding through PASC duration and acute-phase COVID-19 severity. Both univariate and multivariate approaches were employed to investigate the associations between participant characteristics (demographics, PASC duration, acute COVID-19 condition) and OI/POTS/OH prevalences. Statistical significance was defined as *P* < 0.05 for all inferential tests.

### 2.7 Publication bias

Funnel plots were generated to assess potential small-study effects in the meta-analysis. These graphical displays present prevalence estimates on the horizontal axis vs. standard error on the vertical axis. Plot asymmetry was statistically evaluated using Egger's linear regression test and Begg's test. It should be emphasized that conventional publication bias assessment tools, including funnel plot interpretation, were primarily validated for comparative studies analyzing effect sizes with corresponding significance thresholds, rather than for observational studies involving single-arm proportion estimates.

### 2.8 GRADE evaluation

The certainty of evidence was systematically assessed using the GRADEpro Guideline Development Tool (GDT) following the Grading of Recommendations Assessment, Development and Evaluation (GRADE) framework. This evaluation was structured according to five predefined methodological domains (1): study limitations (risk of bias), (2) inconsistency, (3) indirectness, (4) imprecision, and (5) publication bias. Additional domain-specific considerations were incorporated where applicable. Evidence certainty was stratified into four hierarchical levels: high (further research is very unlikely to change confidence in the effect estimate), moderate (further research may have important impact), low (further research is very likely to have important impact), and very low (any effect estimate is uncertain).

## 3 Result

### 3.1 Characteristics of studies included

Two investigators independently conducted a systematic literature search in PubMed, Embase, and CENTRAL, identifying 1,497 preliminary records. Twelve additional grey literature records were retrieved from specialized repositories (e.g., OpenGrey, ProQuest Dissertations). After duplicate removal and a two-phase screening (title/abstract and full-text), 21 studies met the inclusion criteria (Figure 1). Discrepancies were resolved through discussion with a third investigator. The characteristics of included studies are detailed in Tables 1–3. Not all included studies provided complete data on the three predefined clinical endpoints. More specifically, 3 studies recorded the incidence of OI (29–31), 15 studies reported on POTS (17, 18, 31–43), and 12 studies furnished data on the incidence of OH (17, 18, 31, 33–35, 37, 39, 44–47). Furthermore, among the studies investigating OH, only two reported the incidence of initial OH (18, 31), while the remainder documented the incidence of classical OH. The pooled cohort comprised 2,916 participants. Mean participant age ranged 30.0–58.7 years, with mean post-COVID-19 symptom duration spanning 12–72 weeks, and 74.9% of the participants were female. Among the included studies, 19 involved patients with mild COVID-19 cases, only one included moderate COVID-19 cases, and one study comprised patients with severe COVID-19 cases. The baseline demographic and clinical characteristics of the 21 included studies evaluating OI, POTS, and OH are systematically summarized in Tables 1–3.

**Table 1:** Overview of studies included in the meta-analysis on OI.

Reference	Country	Study design	Sample size (female %)	Mean age (years)	Measurement tool	Prevalence data	Acute Covid condition	Post-COVID-19 duration
Eldokla et al. (29)	USA	cross-sectional	322 (73%)	35.9 ± 11.9	COMPASS-31	73.6%	mild	41.9 weeks
Larsen et al. (30)	USA	cross-sectional	1,249 (87.3%)	47.7 ± 11.8	COMPASS-31	68.6%	mild	24 weeks
Hira et al. (31)	Canada	cross-sectional	70 (80%)	43.4 ± 6.0	COMPASS-31	71%	mild	57 weeks

OI: orthostatic intolerance; COMPASS-31: the Composite Autonomic Symptom Score.

**Table 2:** Overview of studies included in the meta-analysis on POTS.

Reference	Country	Study design	Sample size (female %)	Mean age (years)	Measurement tool	Prevalence data	Acute Covid condition	Post-COVID-19 duration
Azcue et al. (32)	Spain	case control	87 (71.3%)	45.6 ± 9.4	HUT	13.8%	mild	55 weeks
Shouman et al. (33)	USA	retrospective	27 (59%)	51 ± 10.3	HUT	22%	mild	17 weeks
Blitshteyn and Whitelaw (34)	USA	case series	20 (70%)	40 ± 10	HUT/AST	75%	mild	24 weeks
Jamal et al. (17)	USA	prospective cohort	24 (83.3%)	43.1 ± 11.3	HUT	16.7%	mild	24weeks
Campen and Visser (35)	Netherlands	cross-sectional	29 (76%)	39 ± 12	HUT	45%	mild	52 weeks
Campen et al. (36)	Netherlands	case control	10 (70%)	30 ± 7	HUT	100%	mild	52 weeks
Demko et al. (37)	USA	prospective cohort	34 (62%)	55 ± 14.8	HUT	5.9%	mild	72 weeks
Hira et al. (31)	Canada	cross-sectional	70 (80%)	43.4 ± 6.0	HUT	30%	mild	57 weeks
Howick et al. (38)	USA	retrospective cohort	363 (58%)	54.3 ± 16.9	HUT	9.1%	mild	48 weeks
Kumar et al. (39)	India	cross-sectional	113 (42.5%)	43 ± 14	HUT	15.0%	moderate	52 weeks
Antonio 2023	Mexico	prospective cohort	23 (60%)	51.1 ± 10.7	HUT	30.4%	mild	43 weeks
Seeley et al. (41)	Australia	prospective cohort	33 (81.8%)	37 ± 15	HUT	79%	mild	25weeks
Campen and Visser (35)	Netherlands	case control	14 (86%)	34 ± 10	HUT	100%	mild	52 weeks
Wang et al. (43)	USA	cross-sectional	126 (66%)	44 ± 15	AST	2.3%	mild	21 weeks
Monaghan et al. (18)	Ireland	cross-sectional	85 (74%)	46 ± 10.2	HUT	8.2%	mild	43 weeks

POTS, postural orthostatic tachycardia syndrome; HUT, head-up tilt table; AST, active standing test.

**Table 3:** Overview of studies included in the meta-analysis on OH.

Reference	Country	Study design	Sample size (female %)	Mean age (years)	Measurement tool	Prevalence data	Acute Covid condition	Post-COVID-19 duration
Salem et al. (44)	Saudi Arabia	case control	28 (35.7%)	30.3 ± 5	HUT	39.3%	mild	24 weeks
Shouman et al. (33)	USA	retrospective	27 (59%)	51 ± 10.3	HUT	11%	mild	17 weeks
Aykaç et al. (45)	Turkey	cross-sectional	56 (57.1%)	41.92 ± 7.91	HUT	17.9%	mild	16 weeks
Blitshteyn and Whitelaw (34)	USA	case series	20 (70%)	40 ± 10	HUT/AST	10%	mild	24 weeks
Jamal et al. (17)	USA	prospective cohort	24 (83.3%)	43.1 ± 11.3	HUT	4.1%	mild	24weeks
Stella 2022	Italy	prospective cohort	180 (70.6%)	51 ± 13	HUT	13.8%	mild	13 weeks
Campen and Visser (35)	Netherlands	cross-sectional	29 (76%)	39 ± 12	HUT	17.2%	mild	52 weeks
Demko et al. (37)	USA	prospective cohort	34 (62%)	55 ± 14.8	HUT	2.9%	mild	72 weeks
Hira et al. (31)	Canada	cross-sectional	70 (80%)	43.4 ± 6.0	HUT	64.3%	mild	57 weeks
Kumar et al. (39)	India	cross-sectional	113 (42.5%)	43 ± 14	HUT	11.5%	moderate	52 weeks
Rass et al. (47)	Austria	prospective cohort	31 (23%)	58.7 ± 9.3	HUT	3%	severe	12 weeks
Monaghan et al. (18)	Ireland	cross-sectional	85 (74%)	46 ± 10.2	HUT	32.9%	mild	43 weeks

OH, orthostatic hypotension; HUT, head-up tilt table; AST, active standing test.

### 3.2 Prevalence of OI

**Figure 2:**
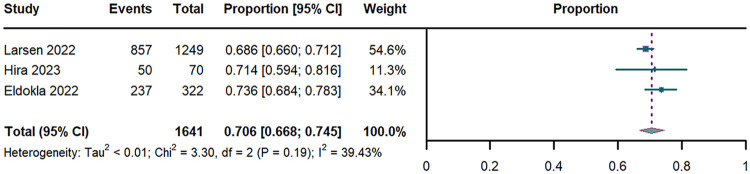
Forest plot of OI prevalence. OI: orthostatic intolerance; CI: confidence interval; df: degree of freedom.

Pooled analysis of 3 studies (*n* = 1,641) (29–31) revealed an overall OI prevalence was 70.6% (95% CI: 66.8%–74.5%; I^2^ = 39.43%; Figure 2).

### 3.3 Prevalence of POTS

**Figure 3:**
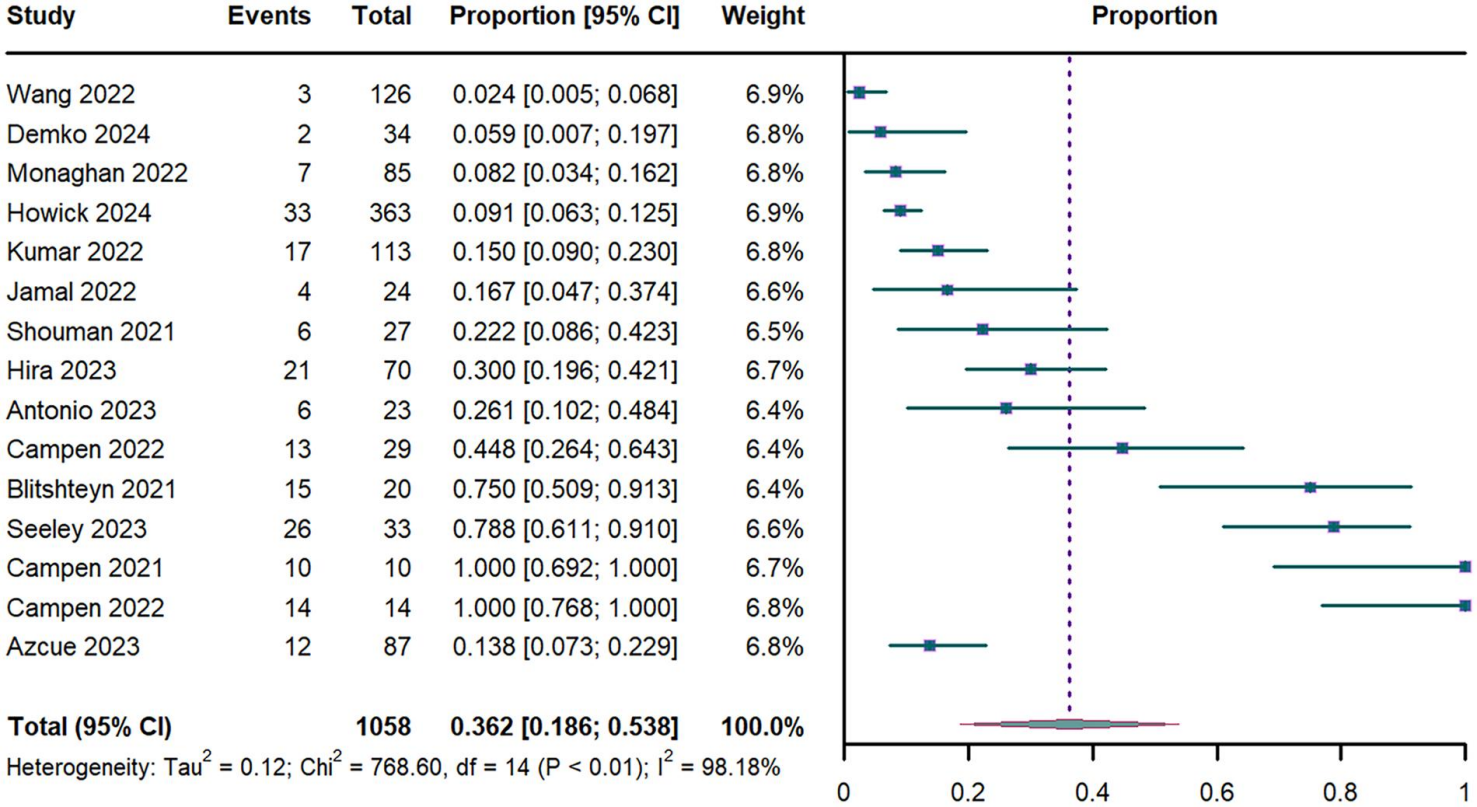
Forest plot of POTS prevalence. POTS: postural orthostatic tachycardia syndrome; CI: confidence interval; df: degree of freedom.

Pooled analysis of 15 studies (*n* = 1,058) (17, 18, 31–43) revealed an overall POTS prevalence of 36.2% (95% CI: 18.6%–53.8%; I^2^ = 98.18%; Figure 3). Stratified analyses showed duration-dependent increases: 29.1% (95% CI: 8.5%–49.8%) for PASC <52 weeks vs. 44.1% (95% CI: 14.2%–73.9%) for ≥52 weeks (Supplementary Figure S1). Prevalence differed significantly by acute-phase severity: 37.8% (95% CI: 19.1%–56.4%) in mild vs. 15% (95% CI: 9%–23%) in moderate COVID-19 cases (Supplementary Figure S2). Leave-one-out Sensitivity analysis confirmed estimate stability (range of 31%–39%; Supplementary Figure S6).

Univariate meta-regression revealed that advancing age was significant negative associated with POTS prevalence (*P* < 0.001; R^2^ = 0.664; Supplementary Table S3 and Figure S8). This association persisted in multivariable models adjusted for sex and PASC duration (*P* = 0.002; R^2^ = 0.667; Supplementary Table S3). In contrast, neither the proportion of female participants (*P* = 0.083; Supplementary Table S3) nor PASC duration (*P*=0.875; Supplementary Table S3) showed significant associations with POTS prevalence.

### 3.4 Prevalence of OH

**Figure 4:**
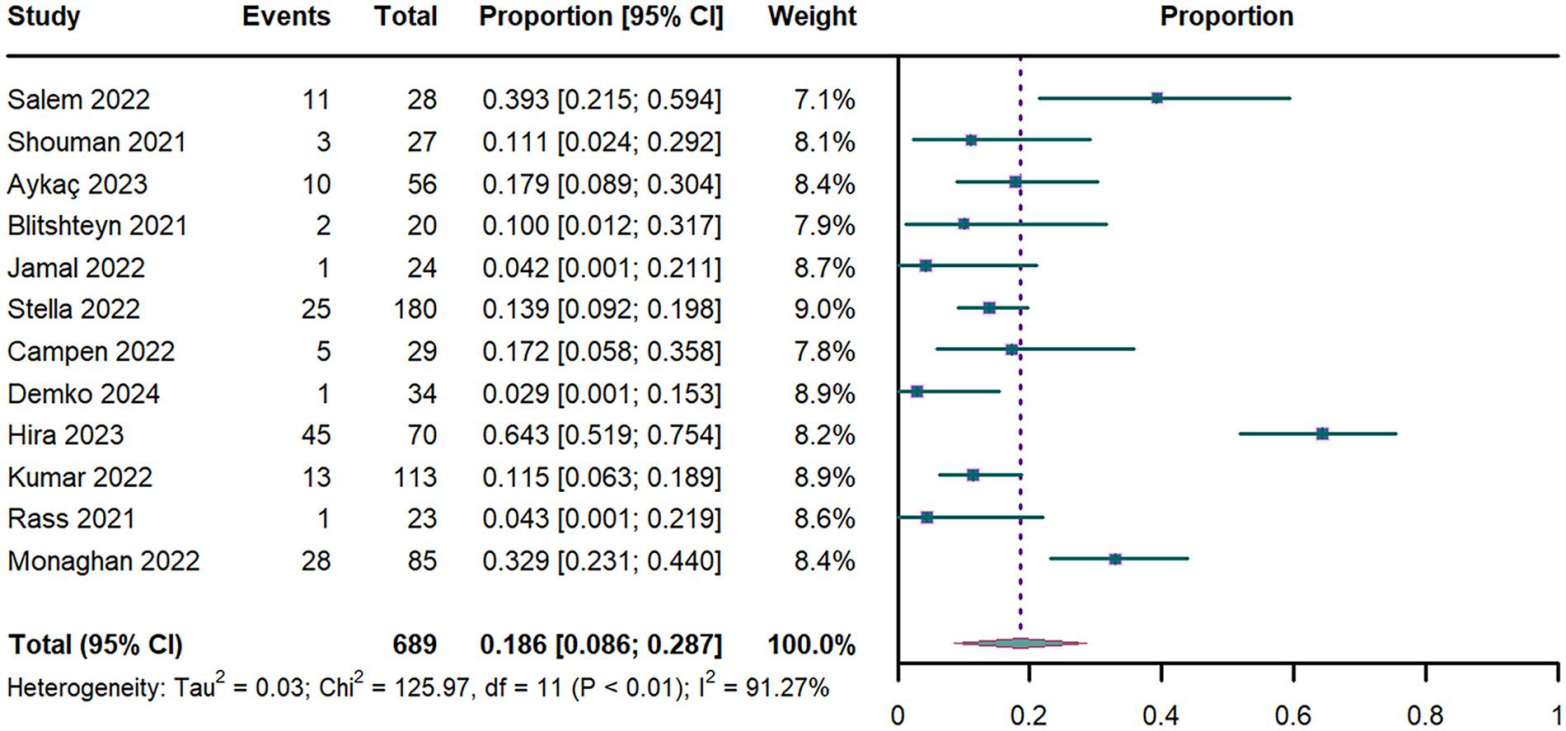
Forest plot of OH prevalence. OH: orthostatic hypotension; CI: confidence interval; df: degree of freedom.

The meta-analysis of 12 studies (*n* = 689) (17, 18, 31, 33–35, 37, 39, 44–47) yielded an OH prevalence of 18.6% (95% CI: 8.6%–28.7%; I^2^ = 91.27%; Figure 4). Temporal stratification revealed lower prevalence in early PASC (15.7% [95% CI: 7.6%–23.8%] for <52 weeks vs. 23.8% [95% CI: 0–50.6%] for ≥52 weeks; Supplementary Figure S3). Acute-phase severity modified prevalence estimates: 21% (95% CI: 9.2%–32.7%) in mild, 11.5% (95% CI: 6.3%–17.9%) in moderate, and 4.3% (95% CI: 0.1%–21.9%) in severe cases (Supplementary Figure S4). Analysis of OH subtypes demonstrated a prevalence of 9.7% (95% CI: 5.8%–13.5%) for classic OH and 43.5% (95% CI: 8.7%–78.3%) for initial OH (Supplementary Figure S5). Leave-one-out Sensitivity analysis showed limited variability (range of 14%–20%; Supplementary Figure S7).

Univariate meta-regression revealed that advancing age was significant negative associated with OH prevalence (*P* = 0.038; R^2^ = 0.364; Supplementary Table S3 and Figure S9). This association persisted in multivariable models adjusted for sex and PASC duration (*P* = 0.011; R^2^ = 0.469; Supplementary Table S3). In contrast, neither the proportion of female participants (*P* = 0.959; Supplementary Table S3) nor PASC duration (*P* = 0.651; Supplementary Table S3) showed significant associations with OH prevalence.

### 3.5 Publication bias

**Figure 5:**
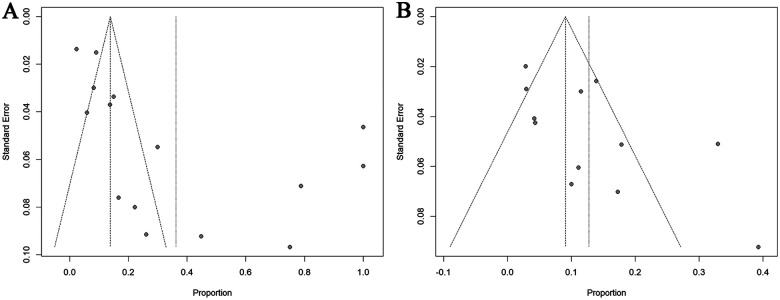
Funnel plot for POTS **(A)** and OH **(B)** prevalence. POTS: postural orthostatic tachycardia syndrome; OH: orthostatic hypotension.

Begg's and Egger's tests are standard methods for assessing publication bias in meta-analyses (48). Begg's test evaluates the association between effect sizes and their sampling variances, with a significant correlation indicating potential publication bias. In contrast, Egger's test employs a linear regression of the standardized effect on precision. A non-significant intercept in this regression suggests the absence of publication bias (48). In this study, both tests found no statistically significant evidence of publication bias for OH. However, results for POTS suggested a potential presence of this bias (Figure 5 and Supplementary Figure S10). The trim-and-fill method was implemented to adjust for this potential bias, estimating that three hypothetical studies would be required to achieve funnel plot symmetry in POTS analyses. Following imputation of these studies using the trim-and-fill algorithm, the adjusted pooled prevalence estimates for POTS decreased from 36.2% to 8.7% (95% CI: 0–30.8%; Supplementary Figure S10).

Notably, single-arm observational studies investigating disease prevalence inherently demonstrate preferential reporting of positive associations between exposures and outcomes. This methodological characteristic suggests that conventional publication bias metrics may have limited interpretative value in prevalence meta-analyses. Specifically, the observed asymmetry could reflect selective outcome reporting rather than true publication bias.

### 3.6 Level of evidence

The certainty of evidence for overall prevalence estimates was rated low for OI and OH due to inconsistencies and risk of bias, and very low for POTS owing to additional publication bias. Despite acknowledged methodological constraints in detecting publication bias within observational study meta-analyses, the certainty of evidence was conservatively downgraded by one level due to residual uncertainty regarding potential publication bias. Domain-specific GRADE evaluations, including detailed rationales for imprecision judgments and subgroup-specific quality assessments, are available in Supplementary Table S4.

## 4 Discussion

Addressing the clinical and public health challenges posed by chronic autonomic dysfunction following COVID-19, as well as informing evidence-based policy formulation, requires robust prevalence estimates among individuals with PASC. Our meta-analysis synthesizes data from 21 studies involving 2,196 individuals, demonstrating an overall prevalence of OI at 70.6% (95% CI: 66.8%–74.5%), POTS at 36.2% (95% CI: 18.6%–53.8%), and OH at 18.6% (95% CI: 8.6%–28.7%). Since the COVID-19 pandemic began in 2019, extrapolating from global estimates of 776 million confirmed COVID-19 cases and a conservative 10% incidence rate for PASC (49), we estimate approximately 54.7 million cases of OI, 28.1 million cases of POTS, and 14.3 million cases of OH worldwide.

The results of our study underscore the substantial public health burden of post-COVID autonomic complications, with their prevalence in long COVID populations exceeding pre-pandemic baselines. Pre-pandemic observational studies reported baseline prevalences of OI at 10.1% (50), POTS at 2% (51) and OH at 6.2% (52) in the general population. However, systematic meta-analyses quantifying OI and POTS prevalence remain scarce. To date, only one indirect meta-analysis has estimated these conditions at 16.9% (OI) and 6.9% (POTS), respectively (53). Notably, no systematic reviews have yet evaluated OI epidemiology during the pandemic period. The sole published meta-analysis reported a POTS incidence of 1.08% among COVID-19 patients—a marked contrast to our findings (54). This discrepancy may stem from methodological limitations. The cited meta-analysis (54) incorporated only five cohort studies, with statistical 94.7% weight assigned to a retrospective cohort by Kwan et al. (55). As explicitly acknowledged in their limitations section, Kwan et al. relied solely on ICD coding for POTS diagnoses rather than standardized diagnostic protocols, which encompassed a substantial proportion of non-POTS cases. The other study was excluded because it enrolled patients whose symptom duration was definitively less than 90 days, thus failing to meet the diagnostic criteria for PASC (56). Consequently, both studies were excluded from our analysis. While numerous cohort studies have investigated OH prevalence, most derive from specialized populations—for instance, reporting 17.7% in type 2 diabetes cohorts (57) and 57.6% in Parkinson's disease patients (58). A meta-analysis synthesizing these data yielded an aggregate OH prevalence of 17%, with subgroup analyses revealing 14% prevalence in non-comorbid populations—a finding concordant with our findings (59). Given the high prevalence of autonomic dysfunction, clinicians should proactively employ tilt-table testing or active standing test to accurately diagnose those conditions, thereby facilitating timely interventions that may mitigate associated quality-of-life impairments and occupational disability linked to autonomic dysregulation (60).

A 3-year longitudinal epidemiological study revealed that the incidence rates of most post-COVID sequelae decline with prolonged disease duration (61). Moreover, a meta-analysis focusing on pulmonary sequelae of COVID-19 demonstrated reduced prevalence rates at 52 weeks post-initial infection compared to 26 weeks (62). However, the prevalence rates of OH and POTS were paradoxically higher in those with disease durations ≥52 weeks than in those with shorter durations. After adjusting for age and sex via multivariate meta-regression analysis, no linear correlation was observed between disease duration and incidence rates. We hypothesize a non-linear temporal relationship between disease duration and the incidence of OH and POTS. Supporting this, a meta-analysis found a higher incidence of post-COVID POTS in patients with disease durations >6 months compared to those with durations of 3–6 months (54). Additionally, a cross-sectional study with a mean follow-up of 23.2 months reported clinical deterioration in a subset of post-COVID POTS patients during longitudinal observation (63).

Through univariate and multivariate meta-regression analysis in our study, a negative correlation was identified between advancing age and the incidence of POTS and OH following COVID-19 infection. This perspective is supported by numerous studies on post-COVID-19 POTS. A systematic review demonstrated that younger individuals with acute COVID-19 exhibited a significantly higher risk of developing persistent autonomic dysfunction compared to older populations (64). Within this cohort, 79% of patients presented with POTS. A systematic review of 21 reports demonstrated that patients developing post-acute COVID-19 POTS were significantly younger (65). Building upon this evidence base, a meta-analysis evaluating post-COVID-19 POTS incidence identified substantially higher prevalence rates among younger populations compared with older adults (54). The findings from studies investigating the relationship between age and OH incidence are inconsistent between the pre-pandemic and post-pandemic periods. Pre-pandemic meta-analyses investigating OH prevalence in primary and institutional care settings have documented a positive correlation with advancing age (59). Notably, these pre-pandemic studies predominantly recruited elderly populations with significant comorbidities, including diabetes mellitus and Parkinson's disease—conditions known to independently contribute to neuropathic autonomic dysfunction. This demographic selection bias may have confounded the observed association between advanced age and orthostatic hypotension susceptibility. A recent systematic review found that young patients with PASC are more prone to developing OH (60).

Current meta-analyses investigating female sex as a risk factor for PASC have yielded conflicting conclusions, which may stem from inadequate adjustment for confounding variables and methodological variations across studies (66, 67). The only published meta-analysis examining the incidence of POTS following COVID-19 is similarly constrained by these limitations (54). That cited analysis stratified studies into two subgroups based on female participation proportion thresholds (>50% vs. ≤50%), conducting stratified meta-regression analyses that revealed no significant association between female sex and POTS incidence (54). To enhance statistical robustness, we implemented inverse-variance weighted meta-regression, operationalizing sex distribution as the proportion of female participants with sample-size adjusted weights. Despite this refined approach, no significant association emerged between female sex and autonomic dysfunction incidence.

Contrary to prior research (61, 68), our meta-analysis challenges the hypothesis that acute COVID-19 severity predicts autonomic dysfunction risk. Notably, our study demonstrates that patients with a mild acute-phase infection were susceptible to POTS and OH. Supporting evidence from a systematic review indicates that among COVID-19 patients with autonomic dysfunction, 67% had mild, 11% moderate, and 22% severe acute infections, respectively (64). This paradoxical pattern may be attributed to the substantial population base of mild COVID-19 cases, which could disproportionately contribute to the epidemiology of post-acute SARS-CoV-2 sequelae despite lower individual risk (69).

The pathophysiological mechanisms are hypothesized to involve autonomic nervous system dysregulation, specifically an imbalance between sympathetic and parasympathetic branches (70). This hypothesis is further substantiated by the work of Wallukat et al., who detected autoantibodies targeting both adrenergic *β*2-receptors and muscarinic M2 receptors in all studied patients following COVID-19 (71). Separately, antibodies against the receptor-binding domain of the SARS-CoV-2 spike protein from COVID-19 patient sera have also demonstrated cross-reactivity with angiotensin II, a primary hormone responsible for blood pressure regulation in humans (72). Collectively, these intricate mechanisms necessitate a multidisciplinary and integrated approach to managing long COVID and its associated autonomic dysfunction. This approach is crucial for understanding the condition's wide-ranging symptoms and their profound impact on patients' daily lives.

To our knowledge, this study represents the first systematic review and meta-analysis that estimates the incidence of predominant autonomic dysfunction phenotypes in PASC. Through comprehensive searches across published and unpublished international databases with strict adherence to predefined inclusion criteria, we achieved robust thematic representation and enhanced statistical precision. The implementation of rigorous methodological standards ensured the consistency and reliability of our results. Furthermore, univariate and multivariate meta-regression analyses identified a significant association between younger age and heightened susceptibility to post-COVID autonomic dysfunction, necessitating prioritized clinical consideration.

Several limitations warrant explicit consideration. First, the predominance of single-center investigations, combined with inconsistent application of randomized sampling methodologies, introduces significant selection bias and elevated statistical heterogeneity. Second, key epidemiological parameters—specifically viral variant stratification, reinfection frequency, and vaccination status, were inadequately documented across included studies. This gap precludes robust assessment of emerging evidence indicating attenuated long COVID risk associated with Omicron variants compared to prior variants of concern (RR: 0.66, 95% CI: 0.61–0.71) (73), potential reinfection-driven symptom exacerbation (HR: 2.10, 95% CI: 2.04–2.16) (74), and prophylactic benefits of vaccination (HR: 0.54, 95% CI: 0.44–0.67) (75) or early antiviral therapy (HR: 0.62, 95% CI: 0.57–0.68) (76). Third, self-reporting instruments in minority of included studies may overestimate true prevalence. When integrated with significant heterogeneity, GRADE assessment yields low to moderate confidence in phenotype-specific risk estimates.

## 5 Conclusion

This meta-analysis reveals that OI, POTS, and OH are prevalent among individuals with PASC, with pooled prevalence rates of 70.6%, 36.2%, and 18.6%, respectively. A significant negative association was observed between advancing age and the incidence of these conditions, while non-linear temporal relationship between PASC duration and the incidence of OH and POTS. These findings underscore the imperative for public health policies prioritizing enhanced screening protocols and standardized diagnostic frameworks for post-COVID autonomic dysfunction.

## Data Availability

The datasets presented in this study can be found in online repositories. The names of the repository/repositories and accession number(s) can be found in the article/Supplementary Material.

## References

[1] World Health Organization. Number of COVID-19 cases reported to WHO (2024). Available online at: https://data.who.int/dashboards/covid19/cases?n=c (Accessed June 15, 2024).

[2] Drake TM Riad AM Fairfield CJ Egan C Knight SR Pius R Characterisation of in-hospital complications associated with COVID-19 using the ISARIC WHO clinical characterisation protocol UK: a prospective, multicentre cohort study. Lancet. (2021) 398(10296):223–37. 10.1016/s0140-6736(21)00799-6 34274064 PMC8285118

[3] Ellul MA Benjamin L Singh B Lant S Michael BD Easton A Neurological associations of COVID-19. Lancet Neurol. (2020) 19(9):767–83. 10.1016/s1474-4422(20)30221-0 32622375 PMC7332267

[4] Bruchfeld A. The COVID-19 pandemic: consequences for nephrology. Nat Rev Nephrol. (2021) 17(2):81–2. 10.1038/s41581-020-00381-4 33257872 PMC7703720

[5] Basso C Leone O Rizzo S De Gaspari M van der Wal AC Aubry MC Pathological features of COVID-19-associated myocardial injury: a multicentre cardiovascular pathology study. Eur Heart J. (2020) 41(39):3827–35. 10.1093/eurheartj/ehaa664 32968776 PMC7543528

[6] Weckbach LT Curta A Bieber S Kraechan A Brado J Hellmuth JC Myocardial inflammation and dysfunction in COVID-19-associated myocardial injury. Circ Cardiovasc Imaging. (2021) 14(1):e012220. 10.1161/circimaging.120.011713 33463366

[7] Lee C Greenwood DC Master H Balasundaram K Williams P Scott JT Prevalence of orthostatic intolerance in long COVID clinic patients and healthy volunteers: a multicenter study. J Med Virol. (2024) 96(3):e29486. 10.1002/jmv.29486 38456315

[8] Huang C Huang L Wang Y Li X Ren L Gu X 6-month Consequences of COVID-19 in patients discharged from hospital: a cohort study. Lancet. (2023) 401(10393):e21–33. 10.1016/S0140-6736(23)00810-3 37321233 PMC10258565

[9] Davis HE Assaf GS McCorkell L Wei H Low RJ Re'em Y Characterizing long COVID in an international cohort: 7 months of symptoms and their impact. EClinicalMedicine. (2021) 38:101019. 10.1016/j.eclinm.2021.101019 34308300 PMC8280690

[10] Groff D Sun A Ssentongo AE Ba DM Parsons N Poudel GR Short-term and long-term rates of postacute sequelae of SARS-CoV-2 infection: a systematic review. JAMA Netw Open. (2021) 4(10):e2128568. 10.1001/jamanetworkopen.2021.28568 34643720 PMC8515212

[11] Brignole M Moya A de Lange FJ Deharo JC Elliott PM Fanciulli A 2018 ESC guidelines for the diagnosis and management of syncope. Eur Heart J. (2018) 39(21):1883–948. 10.1093/eurheartj/ehy037 29562304

[12] Saedon NI Pin Tan M Frith J. The prevalence of orthostatic hypotension: a systematic review and meta-analysis. J Gerontol A Biol Sci Med Sci. (2020) 75(1):117–22. 10.1093/gerona/gly188 30169579 PMC6909901

[13] Mahdi A Lodin K Reistam U Fedorowski A Nygren-Bonnier M Runold M Microvasular dysfunction and reduced cardiac stress reactivity in postural orthostatic tachycardia associated with postacute COVID-19. Circ Arrhythm Electrophysiol. (2023) 16(7):413–4. 10.1161/circep.123.011881 37334702

[14] Rouch L Vidal JS Hoang T Cestac P Hanon O Yaffe K. Systolic blood pressure postural changes variability is associated with greater dementia risk. Neurology. (2020) 95(14):e1932–e40. 10.1212/wnl.0000000000010420 32690802 PMC7682838

[15] Wells R Malik V Brooks AG Linz D Elliott AD Sanders P Cerebral blood flow and cognitive performance in postural tachycardia syndrome: insights from sustained cognitive atress test. J Am Heart Assoc. (2020) 9(24):e017861. 10.1161/jaha.120.017861 33280488 PMC7955388

[16] Tsai Owens MS Biggs BK Fahrenkamp AC Geske J Hofschulte DR Harbeck-Weber C Physical symptoms, distress, and functional disability in youth with chronic orthostatic intolerance. J Pediatr Psychol. (2022) 47(10):1185–94. 10.1093/jpepsy/jsac052 35699566 PMC9960074

[17] Jamal SM Landers D Turi ZG Hollenberg SM Glotzer TV Tancredi J Neurocardiogenic syncope during head-up tilt table (HUTT) testing in patients with post-acute sequela of COVID-19 (PASC): a prospective evaluation. J Am Coll Cardiol. (2022) 79(9):2115. 10.1016/S0735-1097(22)03106-0 PMC8976261 35381331

[18] Monaghan A Jennings G Xue F Byrne L Duggan E Romero-Ortuno R. Orthostatic intolerance in adults reporting long COVID symptoms was not associated with postural orthostatic tachycardia syndrome. Front Physiol. (2022) 13:833650. 10.3389/fphys.2022.833650 35309052 PMC8931464

[19] Page MJ McKenzie JE Bossuyt PM Boutron I Hoffmann TC Mulrow CD The PRISMA 2020 statement: an updated guideline for reporting systematic reviews. Br Med J. (2021) 372:n71. 10.1136/bmj.n71 33782057 PMC8005924

[20] Ely EW Brown LM Fineberg HV. Long COVID defined. N Engl J Med. (2024) 391(18):1746–53. 10.1056/NEJMsb2408466 39083764 PMC11687645

[21] World Health Organization. Clinical management of COVID-19: living guideline, 18 August 2023. Geneva: World Health Organization (2023). (WHO/2019-nCoV/clinical/2023.2). Available online at: https://www.who.int/publications/i/item/WHO-2019-nCoV-clinical-2023.2

[22] Williams EL Raj SR Schondorf R Shen WK Wieling W Claydon VE. Salt supplementation in the management of orthostatic intolerance: vasovagal syncope and postural orthostatic tachycardia syndrome. Auton Neurosci. (2022) 237:102906. 10.1016/j.autneu.2021.102906 34823150

[23] Raj SR Guzman JC Harvey P Richer L Schondorf R Seifer C Canadian Cardiovascular society position statement on postural orthostatic tachycardia syndrome (POTS) and related disorders of chronic orthostatic intolerance. Can J Cardiol. (2020) 36(3):357–72. 10.1016/j.cjca.2019.12.024 32145864

[24] Kim MJ Farrell J. Orthostatic hypotension: a practical approach. Am Fam Physician. (2022) 105(1):39–49. 35029940

[25] Cumpston MS McKenzie JE Welch VA Brennan SE. Strengthening systematic reviews in public health: guidance in the cochrane handbook for systematic reviews of interventions, 2nd edition. J Public Health (Oxf). (2022) 44(4):e588–e92. 10.1093/pubmed/fdac036 35352103 PMC9715291

[26] Munn Z Moola S Lisy K Riitano D Tufanaru C. Chapter 5: Systematic reviews of prevalence and incidence. In: Aromataris E Munn Z, editors. JBI Manual for Evidence Synthesis. Adelaide JBI (2020). p. 1–7.

[27] Schwarzer G Chemaitelly H Abu-Raddad LJ Rücker G. Seriously misleading results using inverse of freeman-tukey double arcsine transformation in meta-analysis of single proportions. Res Synth Methods. (2019) 10(3):476–83. 10.1002/jrsm.1348 30945438 PMC6767151

[28] van Aert RCM Jackson D. A new justification of the hartung-knapp method for random-effects meta-analysis based on weighted least squares regression. Res Synth Methods. (2019) 10(4):515–27. 10.1002/jrsm.1356 31111673 PMC6973024

[29] Eldokla AM Mohamed-Hussein AA Fouad AM Abdelnaser MG Ali ST Makhlouf NA Prevalence and patterns of symptoms of dysautonomia in patients with long-COVID syndrome: a cross-sectional study. Ann Clin Transl Neurol. (2022) 9(6):778–85. 10.1002/acn3.51557 35393771 PMC9110879

[30] Larsen NW Stiles LE Shaik R Schneider L Muppidi S Tsui CT Characterization of autonomic symptom burden in long COVID: a global survey of 2,314 adults. Front Neurol. (2022) 13:1012668. 10.3389/fneur.2022.1012668 36353127 PMC9639503

[31] Hira R Baker JR Siddiqui T Ranada SI Soroush A Karalasingham K Objective hemodynamic cardiovascular autonomic abnormalities in post-acute sequelae of COVID-19. Can J Cardiol. (2023) 39(6):767–75. 10.1016/j.cjca.2022.12.002 36509178 PMC9733966

[32] Azcue N Del Pino R Acera M Fernández-Valle T Ayo-Mentxakatorre N Pérez-Concha T Dysautonomia and small fiber neuropathy in post-COVID condition and chronic fatigue syndrome. J Transl Med. (2023) 21(1):814. 10.1186/s12967-023-04678-3 37968647 PMC10648633

[33] Shouman K Vanichkachorn G Cheshire WP Suarez MD Shelly S Lamotte GJ Autonomic dysfunction following COVID-19 infection: an early experience. Clin Auton Res. (2021) 31(3):385–94. 10.1007/s10286-021-00803-8 33860871 PMC8050227

[34] Blitshteyn S Whitelaw S. Postural orthostatic tachycardia syndrome (POTS) and other autonomic disorders after COVID-19 infection: a case series of 20 patients. Immunol Res. (2021) 69(2):205–11. 10.1007/s12026-021-09185-5 33786700 PMC8009458

[35] Campen C Visser FC. Long-Haul COVID patients: prevalence of POTS are reduced but cerebral blood flow abnormalities remain abnormal with Longer disease duration. Healthcare (Basel. (2022) 10(10):2105. 10.3390/healthcare10102105 36292552 PMC9602558

[36] Campen CLMCV Rowe PC Visser FC. Orthostatic symptoms and reductions in cerebral blood flow in long-haul COVID-19 patients: similarities with myalgic encephalomyelitis/chronic fatigue syndrome. Medicina (kaunas. Lithuania. (2021) 58(1):28. 10.3390/medicina58010028 PMC8778312 35056336

[37] Demko ZO Yu T Mullapudi SK Heslin MGV Dorsey CA Payton CB Two-year longitudinal study reveals that long COVID symptoms peak and quality of life nadirs at 6–12 months postinfection. Open Forum Infect Dis. (2024) 11(3):ofae027. 10.1093/ofid/ofae027 38449921 PMC10917418

[38] Howick JF 5th Saric P Elwazir M Newman DB Pellikka PA Howick AS A pragmatic study of cardiovascular disease during long-term COVID-19. Am J Med. (2025) 138(3):532–40. 10.1016/j.amjmed.2024.03.011 38548213

[39] Kumar NP Thompson S Ramchandra T Waghmare R. Long COVID disease among symptomatic and asymptomatic COVID-19 patients: a multi-centric descriptive study. Eur J Mol Clin Med. (2022) 9(6):1549–55.

[40] González-Hermosillo GJ Galarza EJ Fermín OV González JMN Tostado L Lozano MAE Exaggerated blood pressure elevation in response to orthostatic challenge, a post-acute sequelae of SARS-CoV-2 infection (PASC) after hospitalization. Auton Neurosci. (2023) 247:103094. 10.1016/j.autneu.2023.103094 37137186 PMC10121145

[41] Seeley MC Gallagher C Ong E Langdon A Chieng J Bailey D High incidence of autonomic dysfunction and postural orthostatic tachycardia syndrome in patients with long COVID: implications for management and health care planning. Am J Med. (2023) 138(2):354–361.e1. 10.1016/j.amjmed.2023.06.010 37391116 PMC10307671

[42] van Campen C Visser FC. Orthostatic intolerance in long-haul COVID after SARS-CoV-2: a case-control comparison with post-EBV and insidious-onset myalgic encephalomyelitis/chronic fatigue syndrome patients. Healthcare (Basel). (2022) 10(10):2058. 10.3390/healthcare10102058 36292504 PMC9602265

[43] Wang SY Adejumo P See C Onuma OK Miller EJ Spatz ES. Characteristics of patients referred to a cardiovascular disease clinic for post-acute sequelae of SARS-CoV-2 infection. Am Heart J Plus. (2022) 18:100176. 10.1016/j.ahjo.2022.100176 35856065 PMC9277988

[44] Salem AM Yar T Al Eid M Almahfoudh H Alsaffar M Al Ibrahim A Post-acute effect of SARS-CoV-2 infection on the cardiac autonomic function. Int J Gen Med. (2022) 15:7593–603. 10.2147/IJGM.S382331 36204699 PMC9531620

[45] Aykaç S Büyükşireci DE Boyacı H. An analysis of neuropathic pain, vasomotor manifestations, and sympathetic skin reactions in post-COVID-19 patients relative to healthy individuals. Medicine (United States). (2023) 102(43):E35819. 10.1097/MD.0000000000035819 PMC10615396 37904350

[46] Buoite Stella A Furlanis G Frezza NA Valentinotti R Ajcevic M Manganotti P. Autonomic dysfunction in post-COVID patients with and witfhout neurological symptoms: a prospective multidomain observational study. J Neurol. (2022) 269(2):587–96. 10.1007/s00415-021-10735-y 34386903 PMC8359764

[47] Rass V Beer R Schiefecker AJ Kofler M Lindner A Mahlknecht P Neurological outcome and quality of life 3 months after COVID-19: a prospective observational cohort study. Eur J Neurol. (2021) 28(10):3348–59. 10.1111/ene.14803 33682276 PMC8250725

[48] Lin L Chu H. Quantifying publication bias in meta-analysis. Biometrics. (2018) 74(3):785–94. 10.1111/biom.12817 29141096 PMC5953768

[49] Ballering AV van Zon SKR Olde Hartman TC Rosmalen JGM. Persistence of somatic symptoms after COVID-19 in The Netherlands: an observational cohort study. Lancet. (2022) 400(10350):452–61. 10.1016/s0140-6736(22)01214-4 35934007 PMC9352274

[50] Winker R Barth A Dorner W Mayr O Pilger A Ivancsits S Diagnostic management of orthostatic intolerance in the workplace. Int Arch Occup Environ Health. (2003) 76(2):143–50. 10.1007/s00420-002-0395-4 12733087

[51] Brinth L Pors K Spahic JM Sutton R Fedorowski A Mehlsen J. Postural orthostatic tachycardia syndrome (POTS) in Denmark: increasingly recognized or new epidemic? Auton Neurosci. (2018) 213:92–5. 10.1016/j.autneu.2018.03.001 29530592

[52] Fedorowski A Stavenow L Hedblad B Berglund G Nilsson PM Melander O. Orthostatic hypotension predicts all-cause mortality and coronary events in middle-aged individuals (the malmo preventive project). Eur Heart J. (2010) 31(1):85–91. 10.1093/eurheartj/ehp329 19696189 PMC2800919

[53] Loughlin EA Judge CS Gorey SE Costello MM Murphy RP Waters RF Increased salt intake for orthostatic intolerance syndromes: a systematic review and meta-analysis. Am J Med. (2020) 133(12):1471–8.E4. 10.1016/j.amjmed.2020.05.028 32603788

[54] Yong SJ Halim A Liu S Halim M Alshehri AA Alshahrani MA Pooled rates and demographics of POTS following SARS-CoV-2 infection versus COVID-19 vaccination: systematic review and meta-analysis. Auton Neurosci. (2023) 250:103132. 10.1016/j.autneu.2023.103132 38000119

[55] Kwan AC Ebinger JE Wei J Le CN Oft JR Zabner R Apparent risks of postural orthostatic tachycardia syndrome diagnoses after COVID-19 vaccination and SARS-cov-2 infection. Nat Cardiovasc Res. (2022) 1(12):1187–94. 10.1038/s44161-022-00177-8 37303827 PMC10254901

[56] Shah B Kunal S Bansal A Jain J Poundrik S Shetty MK Heart rate variability as a marker of cardiovascular dysautonomia in post-COVID-19 syndrome using artificial intelligence. Indian Pacing Electrophysiol J. (2022) 22(2):70–6. 10.1016/j.ipej.2022.01.004 35101582 PMC8800539

[57] Bouhanick B Meliani S Doucet J Bauduceau B Verny C Chamontin B Orthostatic hypotension is associated with more severe hypertension in elderly autonomous diabetic patients from the French gerodiab study at inclusion. Ann Cardiol Angeiol (Paris). (2014) 63(3):176–82. 10.1016/j.ancard.2014.05.013 24958527

[58] Hommel A Faber MJ Weerkamp NJ van Dijk JG Bloem BR Koopmans RT. Prevalence and prescribed treatments of orthostatic hypotension in institutionalized patients with Parkinson’s disease. J Parkinsons Dis. (2016) 6(4):805–10. 10.3233/jpd-160853 27662327

[59] McDonagh STJ Mejzner N Clark CE. Prevalence of postural hypotension in primary, community and institutional care: a systematic review and meta-analysis. BMC Fam Pract. (2021) 22(1):1. 10.1186/s12875-020-01313-8 33388038 PMC7777418

[60] Fedorowski A Fanciulli A Raj SR Sheldon R Shibao CA Sutton R. Cardiovascular autonomic dysfunction in post-COVID-19 syndrome: a major health-care burden. Nat Rev Cardiol. (2024) 21(6):379–95. 10.1038/s41569-023-00962-3 38163814

[61] Cai M Xie Y Topol EJ Al-Aly Z. Three-year outcomes of post-acute sequelae of COVID-19. Nat Med. (2024) 30(6):1564–73. 10.1038/s41591-024-02987-8 38816608 PMC11186764

[62] Lee JH Yim JJ Park J. Pulmonary function and chest computed tomography abnormalities 6–12 months after recovery from COVID-19: a systematic review and meta-analysis. Respir Res. (2022) 23(1):233. 10.1186/s12931-022-02163-x 36068582 PMC9446643

[63] Hurt RT Yadav S Schroeder DR Croghan IT Mueller MR Grach SL Longitudinal progression of patients with long COVID treated in a post-COVID clinic: a cross-sectional survey. J Prim Care Community Health. (2024) 15:21501319241258671. 10.1177/21501319241258671 38813984 PMC11141226

[64] Reis Carneiro D Rocha I Habek M Helbok R Sellner J Struhal W Clinical presentation and management strategies of cardiovascular autonomic dysfunction following a COVID-19 infection—a systematic review. Eur J Neurol. (2023) 30(5):1528–39. 10.1111/ene.15714 36694382

[65] Abbate G De Iulio B Thomas G Priday A Biondi-Zoccai G Markley R Postural orthostatic tachycardia syndrome after COVID-19: a systematic review of therapeutic interventions. J Cardiovasc Pharmacol. (2023) 82(1):23–31. 10.1097/fjc.0000000000001432 37094584

[66] Xu Z Wang W Zhang D Tam KW Li Y Chan DCC Excess risks of long COVID symptoms compared with identical symptoms in the general population: a systematic review and meta-analysis of studies with control groups. J Glob Health. (2024) 14:05022. 10.7189/jogh.14.05022 39129538 PMC11317913

[67] Luo D Mei B Wang P Li X Chen X Wei G Prevalence and risk factors for persistent symptoms after COVID-19: a systematic review and meta-analysis. Clin Microbiol Infect. (2024) 30(3):328–35. 10.1016/j.cmi.2023.10.016 37866679

[68] Romero Starke K Kaboth P Rath N Reissig D Kaempf D Nienhaus A Cardiovascular disease risk after a SARS-CoV-2 infection: a systematic review and meta-analysis. J Infect. (2024) 89(3):106215. 10.1016/j.jinf.2024.106215 38971381

[69] Wulf Hanson S Abbafati C Aerts JG Al-Aly Z Ashbaugh C Ballouz T Estimated global proportions of individuals with persistent fatigue, cognitive, and respiratory symptom clusters following symptomatic COVID-19 in 2020 and 2021. JAMA. (2022) 328(16):1604–15. 10.1001/jama.2022.18931 36215063 PMC9552043

[70] Fedorowski A Sutton R. Autonomic dysfunction and postural orthostatic tachycardia syndrome in post-acute COVID-19 syndrome. Nat Rev Cardiol. (2023) 20(5):281–2. 10.1038/s41569-023-00842-w 36732397 PMC9893964

[71] Wallukat G Hohberger B Wenzel K Fürst J Schulze-Rothe S Wallukat A Functional autoantibodies against G-protein coupled receptors in patients with persistent long-COVID-19 symptoms. J Transl Autoimmun. (2021) 4:100100. 10.1016/j.jtauto.2021.100100 33880442 PMC8049853

[72] Briquez PS Rouhani SJ Yu J Pyzer AR Trujillo J Dugan HL Severe COVID-19 induces autoantibodies against angiotensin II that correlate with blood pressure dysregulation and disease severity. Sci Adv. (2022) 8(40):eabn3777. 10.1126/sciadv.abn3777 36206332 PMC9544317

[73] Xie Y Choi T Al-Aly Z. Postacute sequelae of SARS-CoV-2 infection in the pre-Delta, Delta, and omicron eras. N Engl J Med. (2024) 391(6):515–25. 10.1056/NEJMoa2403211 39018527 PMC11687648

[74] Bowe B Xie Y Al-Aly Z. Acute and postacute sequelae associated with SARS-CoV-2 reinfection. Nat Med. (2022) 28(11):2398–405. 10.1038/s41591-022-02051-3 36357676 PMC9671810

[75] Català M Mercadé-Besora N Kolde R Trinh NTH Roel E Burn E The effectiveness of COVID-19 vaccines to prevent long COVID symptoms: staggered cohort study of data from the UK, Spain, and Estonia. Lancet Respir Med. (2024) 12(3):225–36. 10.1016/s2213-2600(23)00414-9 38219763

[76] Wang H Wei Y Hung CT Lin G Jiang X Li C Association of nirmatrelvir-ritonavir with post-acute sequelae and mortality in patients admitted to hospital with COVID-19: a retrospective cohort study. Lancet Infect Dis. (2024) 24(10):1130–40. 10.1016/s1473-3099(24)00217-2 38710190

