# Supplementary material for: The prevalence of orthostatic intolerance, postural orthostatic tachycardia syndrome and orthostatic hypotension in post-acute sequelae of COVID-19
Source: Front Cardiovasc Med. 2026 Jan 8;12:1679252. doi: 10.3389/fcvm.2025.1679252 (PMC12824017; doi:10.3389/fcvm.2025.1679252)
Supplement: Supplementary file 1 [file Datasheet1.doc]

**SUPPLEMENTARY MATERIAL**

Contents

[**Supplementary Table S1:** search strategy 1](#__RefHeading___Toc214809519)

[**Supplementary Table S2:** Quality assessment for including studies. 3](#__RefHeading___Toc214809520)

[**Supplementary Table S3:** Meta-regression showing results of uni- and multivariate models for POTS and OH. 5](#__RefHeading___Toc214809521)

[**Supplementary Table S4:** GRADE Table displaying level of evidence for OI, POTS and OH. 6](#__RefHeading___Toc214809522)

[**Supplementary Figure S1:** Forest plot of POTS prevalence stratified by PASC duration. 7](#__RefHeading___Toc214809523)

[**Supplementary Figure S2:** Forest plot of POTS prevalence stratified by acute Covid condition. 8](#__RefHeading___Toc214809524)

[**Supplementary Figure S3:** Forest plot of OH prevalence stratified by PASC duration. 9](#__RefHeading___Toc214809525)

[**Supplementary Figure S4:** Forest plot of OH prevalence stratified by acute Covid condition. 10](#__RefHeading___Toc214809526)

[**Supplementary Figure S5**: Forest plot of OH prevalence stratified by subtypes. 11](#__RefHeading___Toc214809527)

[**Supplementary Figure S6:** Sensitivity analyses of POTS prevalence. 12](#__RefHeading___Toc214809528)

[**Supplementary Figure S7:** Sensitivity analyses of OH prevalence. 12](#__RefHeading___Toc214809529)

[**Supplementary Figure S8:** Bubble plots of POTS prevalence by mean age of participants. 13](#__RefHeading___Toc214809530)

[**Supplementary Figure S9:** Bubble plots of OH prevalence by mean age of participants. 14](#__RefHeading___Toc214809531)

[**Supplementary Figure S10:** A: Egger's test for publication bias in POTS studies and B: The trim and fill funnel plot for POTS studies. 15](#__RefHeading___Toc214809532)

### **Supplementary Table S1:** search strategy

1.1 Orthostatic intolerance

| **Search** | **Query** |
| --- | --- |
| **#1** | "SARS-CoV-2"[MeSH Terms] OR "COVID-19"[MeSH Terms] OR "Post-Acute COVID-19 Syndrome"[MeSH Terms] OR "2019 ncov"[Title/Abstract] OR "2019 novel coronavirus"[Title/Abstract] OR "COVID-19"[Title/Abstract] OR "new coronavirus"[Title/Abstract] OR "novel coronavirus"[Title/Abstract] OR "SARS-CoV-2"[Title/Abstract] OR "SARS-CoV"[Title/Abstract] OR "coronavirus disease"[Title/Abstract] OR "long covid"[Title/Abstract] OR "post covid"[Title/Abstract] |
| **#2** | "orthostatic intolerance"[MeSH Terms] OR "orthostatic intolerance"[Title/Abstract] OR "postural intolerance"[Title/Abstract] |
| **#3** | "prevalence"[MeSH Terms] OR "cross-sectional studies"[MeSH Terms] OR "epidemiologic studies"[MeSH Terms] OR "case-control studies "[MeSH Terms] OR "cohort studies"[MeSH Terms] OR "prevalence"[Title/Abstract] OR "cross?sectional stud*"[Title/Abstract] OR "case?control"[Title/Abstract] OR "cohort"[Title/Abstract] OR " observational"[Title/Abstract] OR "retrospective"[Title/Abstract] OR "prospective"[Title/Abstract]) |
| **#4** | #1 AND #2 AND #3 |

1.2 Postural orthostatic tachycardia syndrome

| **Search** | **Query** |
| --- | --- |
| **#1** | "SARS-CoV-2"[MeSH Terms] OR "COVID-19"[MeSH Terms] OR "Post-Acute COVID-19 Syndrome"[MeSH Terms] OR "2019 ncov"[Title/Abstract] OR "2019 novel coronavirus"[Title/Abstract] OR "COVID-19"[Title/Abstract] OR "new coronavirus"[Title/Abstract] OR "novel coronavirus"[Title/Abstract] OR "SARS-CoV-2"[Title/Abstract] OR "SARS-CoV"[Title/Abstract] OR "coronavirus disease"[Title/Abstract] OR "long covid"[Title/Abstract] OR "post covid"[Title/Abstract] |
| **#2** | "postural orthostatic tachycardia syndrome"[MeSH Terms] OR "postural orthostatic tachycardia syndrome"[Title/Abstract] OR "postural tachycardia"[Title/Abstract] OR "orthostatic tachycardia"[Title/Abstract] OR "pots"[Title/Abstract] |
| **#3** | "prevalence"[MeSH Terms] OR "cross-sectional studies"[MeSH Terms] OR "epidemiologic studies"[MeSH Terms] OR "case-control studies "[MeSH Terms] OR "cohort studies"[MeSH Terms] OR "prevalence"[Title/Abstract] OR "cross?sectional stud*"[Title/Abstract] OR "case?control"[Title/Abstract] OR "cohort"[Title/Abstract] OR " observational"[Title/Abstract] OR "retrospective"[Title/Abstract] OR "prospective"[Title/Abstract]) |
| **#4** | #1 AND #2 AND #3 |

1.3 Orthostatic hypotension

| **Search** | **Query** |
| --- | --- |
| **#1** | "SARS-CoV-2"[MeSH Terms] OR "COVID-19"[MeSH Terms] OR "Post-Acute COVID-19 Syndrome"[MeSH Terms] OR "2019 ncov"[Title/Abstract] OR "2019 novel coronavirus"[Title/Abstract] OR "COVID-19"[Title/Abstract] OR "new coronavirus"[Title/Abstract] OR "novel coronavirus"[Title/Abstract] OR "SARS-CoV-2"[Title/Abstract] OR "SARS-CoV"[Title/Abstract] OR "coronavirus disease"[Title/Abstract] OR "long covid"[Title/Abstract] OR "post covid"[Title/Abstract] |
| **#2** | "hypotension, orthostatic"[MeSH Terms] OR "orthostatic hypotens*"[Title/Abstract] OR "postural hypotens*"[Title/Abstract] OR "neurally mediated hypotens*"[Title/Abstract] OR "hypotens*"[Title/Abstract]) |
| **#3** | "prevalence"[MeSH Terms] OR "cross-sectional studies"[MeSH Terms] OR "epidemiologic studies"[MeSH Terms] OR "case-control studies "[MeSH Terms] OR "cohort studies"[MeSH Terms] OR "prevalence"[Title/Abstract] OR "cross?sectional stud*"[Title/Abstract] OR "case?control"[Title/Abstract] OR "cohort"[Title/Abstract] OR " observational"[Title/Abstract] OR "retrospective"[Title/Abstract] OR "prospective"[Title/Abstract]) |
| **#4** | #1 AND #2 AND #3 |

### **Supplementary Table S2:** Quality assessment for including studies.

| Study | Was the sample frame appropriate to address the target population? | Were study participants sampled in an appropriate way? | Was the sample size adequate? | Were the study subjects and the setting described in detail? | Was the data analysis conducted with sufficient coverage of the identified sample? | Were valid methods used for the identification of the condition? | Was the condition measured in a standard, reliable way for all participants? | Was there appropriate statistical analysis? | Was the response rate adequate, and if not, was the low response rate managed appropriately? |
| --- | --- | --- | --- | --- | --- | --- | --- | --- | --- |
| Eldokla 2022 | Yes | Yes | Yes | Yes | Yes | No | No | Yes | Yes |
| Larsen 2022 | Yes | Yes | Yes | Yes | Yes | No | No | Yes | No |
| Salem 2022 | No | Unclear | No | Yes | Yes | Yes | Yes | Yes | Yes |
| Shouman 2021 | No | No | No | No | Yes | Yes | Yes | Yes | Yes |
| Aykaç 2023 | Yes | Yes | Yes | Yes | Yes | Yes | Yes | Yes | Yes |
| Azcue 2023 | Yes | Yes | Yes | Yes | Yes | Yes | Yes | Yes | Yes |
| Blitshteyn 2021 | No | No | No | Yes | Yes | Yes | Yes | No | Yes |
| Jamal 2022 | Yes | Yes | No | Yes | No | Yes | Yes | Yes | Yes |
| Stella 2022 | Yes | Yes | Yes | Yes | Yes | Yes | Yes | Yes | Yes |
| Campen 2022 | No | No | No | Yes | No | Yes | Yes | Yes | Yes |
| Campen 2021 | No | No | No | Yes | No | Yes | Yes | Yes | Yes |
| Demko 2024 | Yes | No | No | Yes | Yes | Yes | Yes | Yes | Yes |
| Hira 2023 | Yes | No | Yes | Yes | Yes | Yes | Yes | Yes | Yes |
| Kumar 2022 | Yes | No | Yes | No | Yes | Yes | Yes | Yes | Yes |
| Rass 2021 | No | No | No | Yes | Yes | Yes | No | Yes | Yes |
| Antonio 2023 | No | No | Yes | Yes | Yes | Yes | Yes | Yes | Yes |
| Seeley 2023 | No | No | No | Yes | Yes | Yes | Yes | Yes | Yes |
| Campen 2022 | No | No | No | Yes | Yes | Yes | Yes | Yes | Yes |
| Wang 2022 | Yes | Yes | Yes | Yes | Yes | No | Yes | Yes | Yes |
| Monaghan 2022 | Yes | Yes | Yes | Yes | Yes | Yes | Yes | Yes | Yes |
| Howick 2024 | Yes | Yes | Yes | Yes | Yes | Yes | Yes | Yes | Yes |

### **Supplementary Table S3:** Meta-regression showing results of uni- and multivariate models for POTS and OH.

|  | POTS | | OH | |
| --- | --- | --- | --- | --- |
|  | Univariate | Multivariate | Univariate | Multivariate |
| Mean age | Estimate= -0.039  P < 0.001  F＝25.71  R^2^=0.664 | Estimate= -0.037  P = 0.002  F＝7.358  R^2^=0.667 | Estimate= -0.091  p＝0.038  F＝5.72  R^2^=0.364 | Estimate= -0.007  p＝0.011  F＝2.363  R^2^ =0.469 |
| proportion of female | NS  (p=0.083) | NS  (p=0.782) | NS  (p=0.959) | NS  (p=0.421) |
| PASC duration | NS  (p=0.875) | NS  (p=0.855) | NS  (p=0.651) | NS  (p=0.591) |

POTS: postural orthostatic tachycardia syndrome; OH: orthostatic hypotension; NS: Non-Significant.

### **Supplementary Table S4:** GRADE Table displaying level of evidence for OI, POTS and OH.

| **№ of studies** | **Certainty assessment** | | | | | | **Effect** | | | **Certainty** | **Importance** |
| --- | --- | --- | --- | --- | --- | --- | --- | --- | --- | --- | --- |
|  | **Study design** | **Risk of bias** | **Inconsistency** | **Indirectness** | **Imprecision** | **Other considerations** | **№ of events** | **№ of individuals** | **Rate (95% CI)** |  |  |
| prevalence of OI (follow-up: range 24 weeks to 57 weeks) | | | | | | | | | | | |
| 3 | non-randomised studies | serious^a^ | serious^b^ | not serious | not serious | none | 1144 | 1641 | event rate 706 per 1000 (0.668 to 0.745) | ⨁⨁◯◯ Low^a,b^ | IMPORTANT |
| prevalence of POTS (follow-up: range 17 weeks to 72 weeks) | | | | | | | | | | | |
| 15 | non-randomised studies | serious^a^ | serious^b^ | not serious | not serious | publication bias strongly suspected^c^ | 189 | 1058 | event rate 362 per 1000 (0.186 to 0.538) | ⨁◯◯◯ Very low^a,b,c^ | NOT IMPORTANT |
| prevalence of OH (follow-up: range 12 weeks to 72 weeks) | | | | | | | | | | | |
| 12 | non-randomised studies | serious^a^ | serious^b^ | not serious | not serious | none | 102 | 689 | event rate 186 per 1000 (0.086 to 0.287) | ⨁⨁◯◯ Low^a,b^ | IMPORTANT |

OI: orthostatic intolerance; POTS: postural orthostatic tachycardia syndrome; OH: orthostatic hypotension.

#### Explanations

a. Several studies exhibit methodological limitations, including non-representative sampling frameworks, inadequate characterization of study cohorts, and utilization of non-validated assessment instruments, which may compromise the reliability and generalizability of findings.

b. High I² (inconsistency) in meta-analysis. The proportion of variability explained by the meta-regression analysis (R-squared value) is not very high.

c. The Begg's test suggested a potential presence of publication bias.

### **Supplementary Figure S1:** Forest plot of POTS prevalence stratified by PASC duration.

**
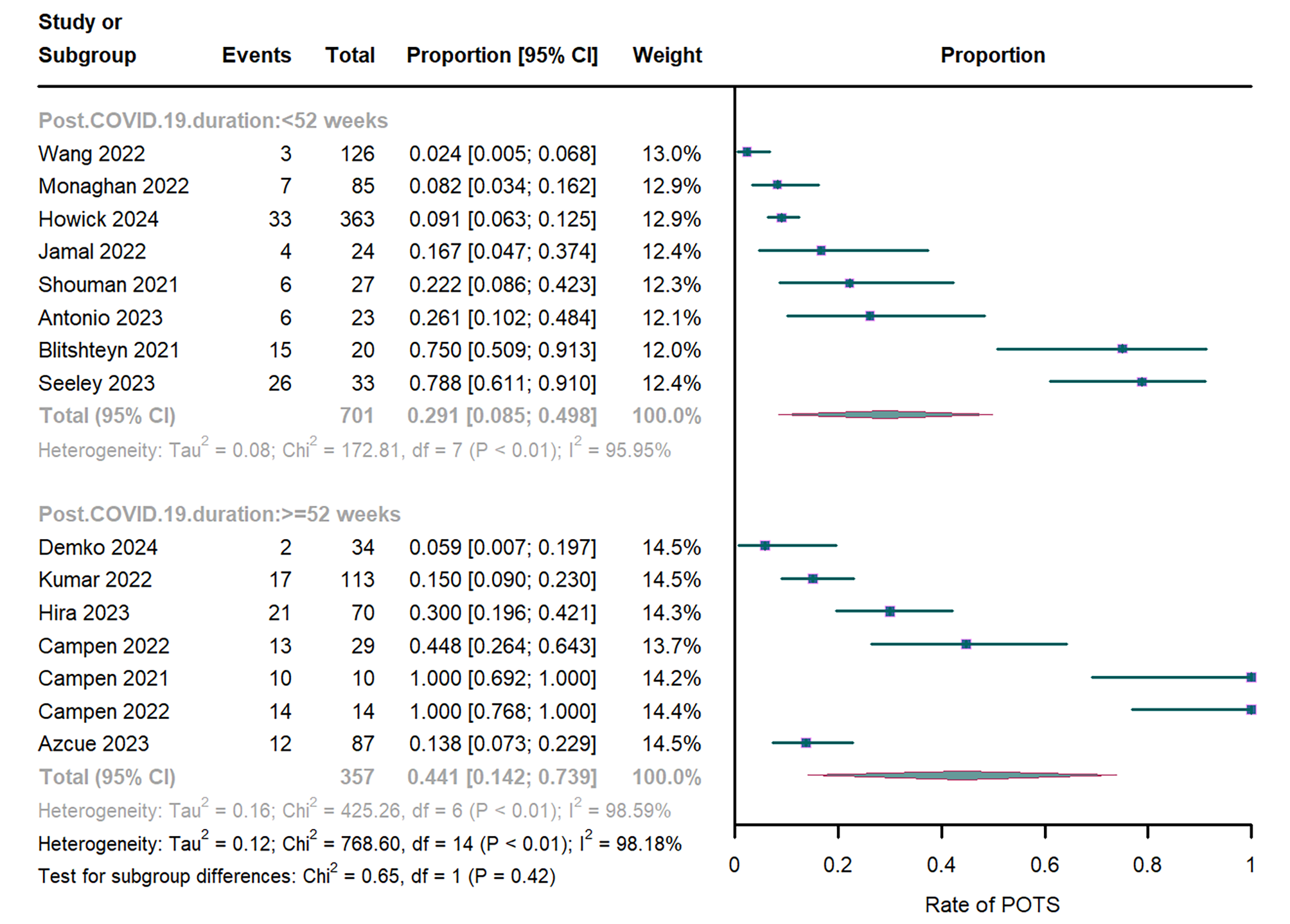
**

### **Supplementary Figure S2:** Forest plot of POTS prevalence stratified by acute Covid condition.

**
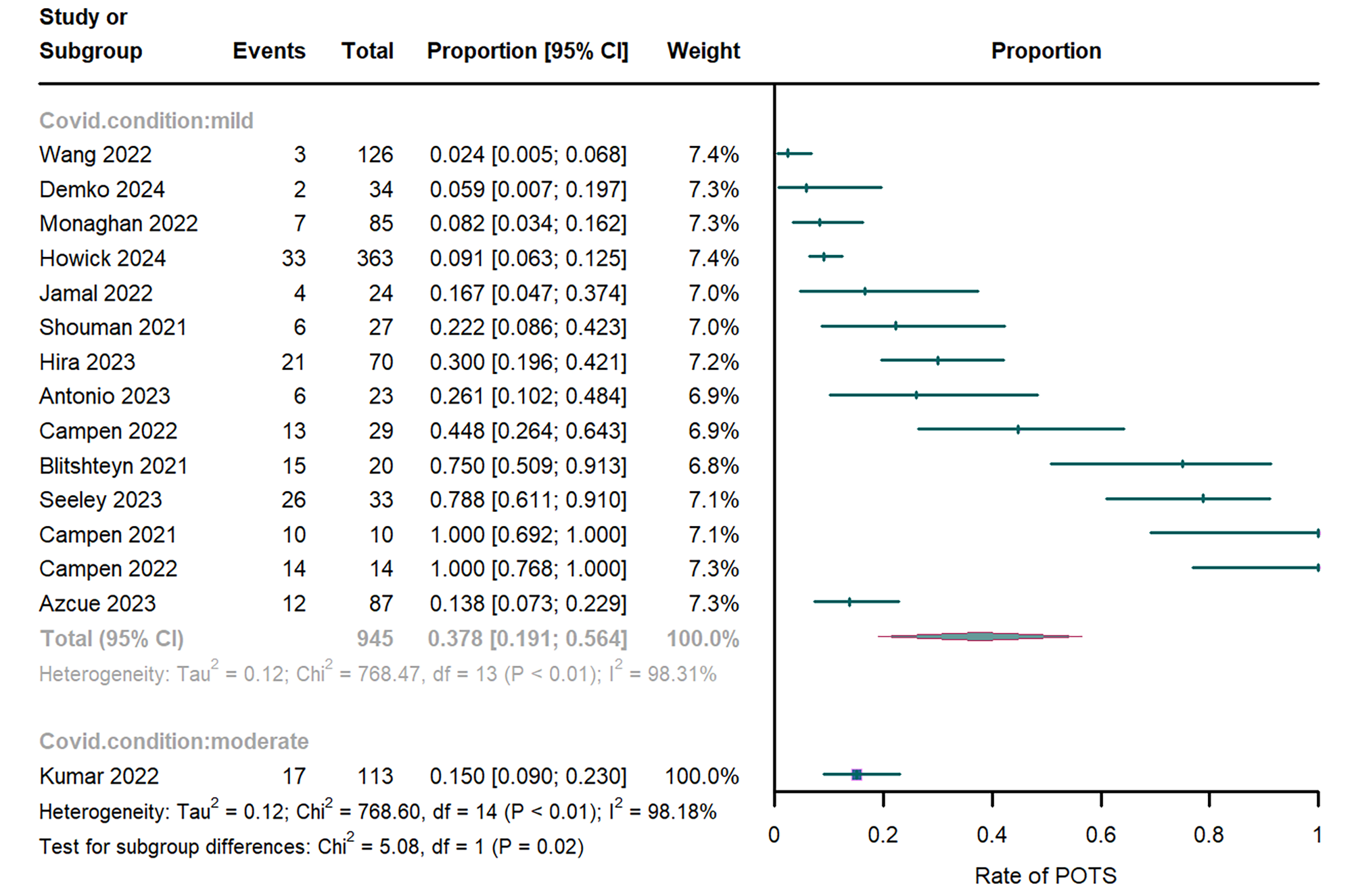
**

### **Supplementary Figure S3:** Forest plot of OH prevalence stratified by PASC duration.

**
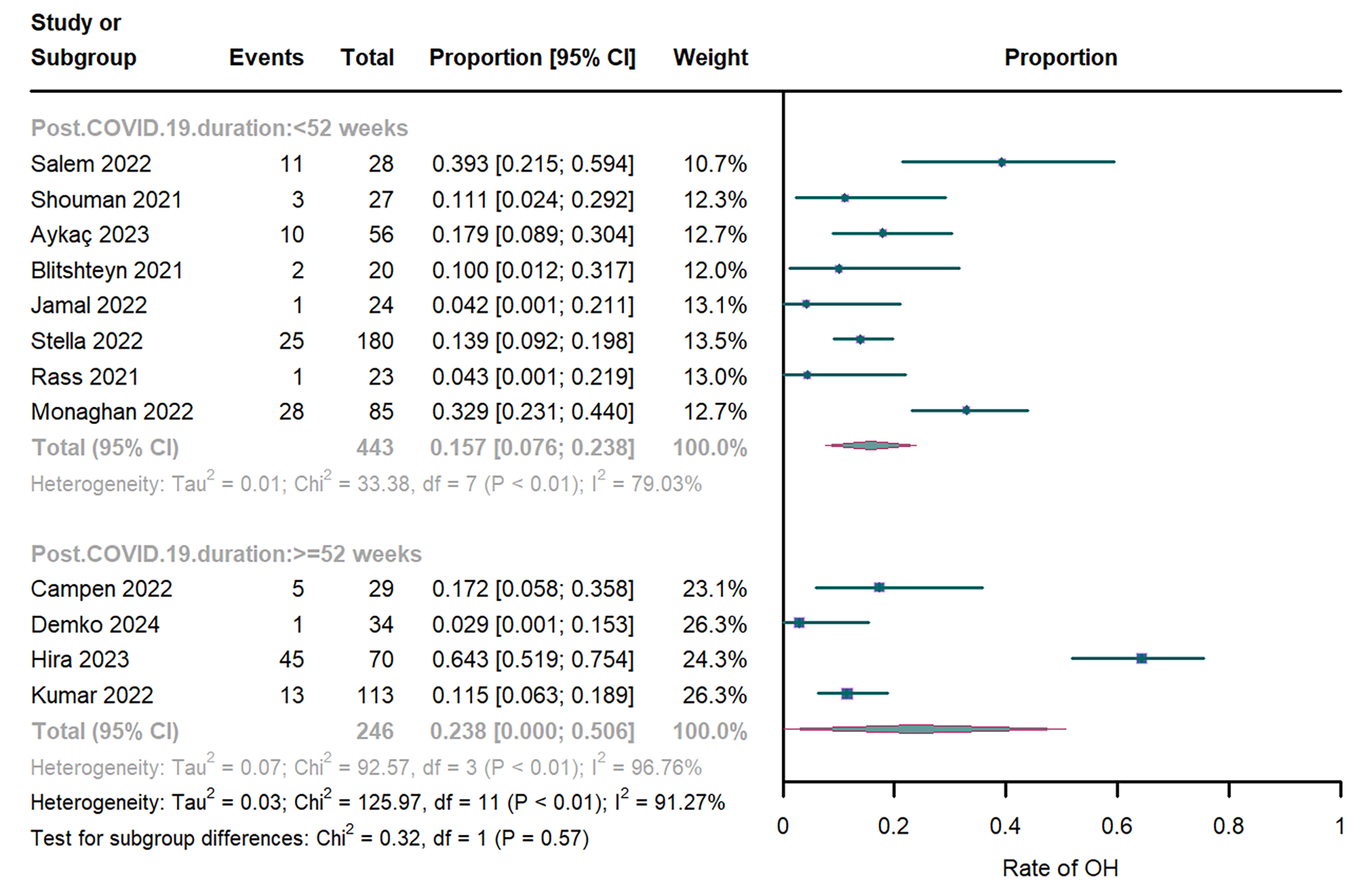
**

### **Supplementary Figure S4:** Forest plot of OH prevalence stratified by acute Covid condition.


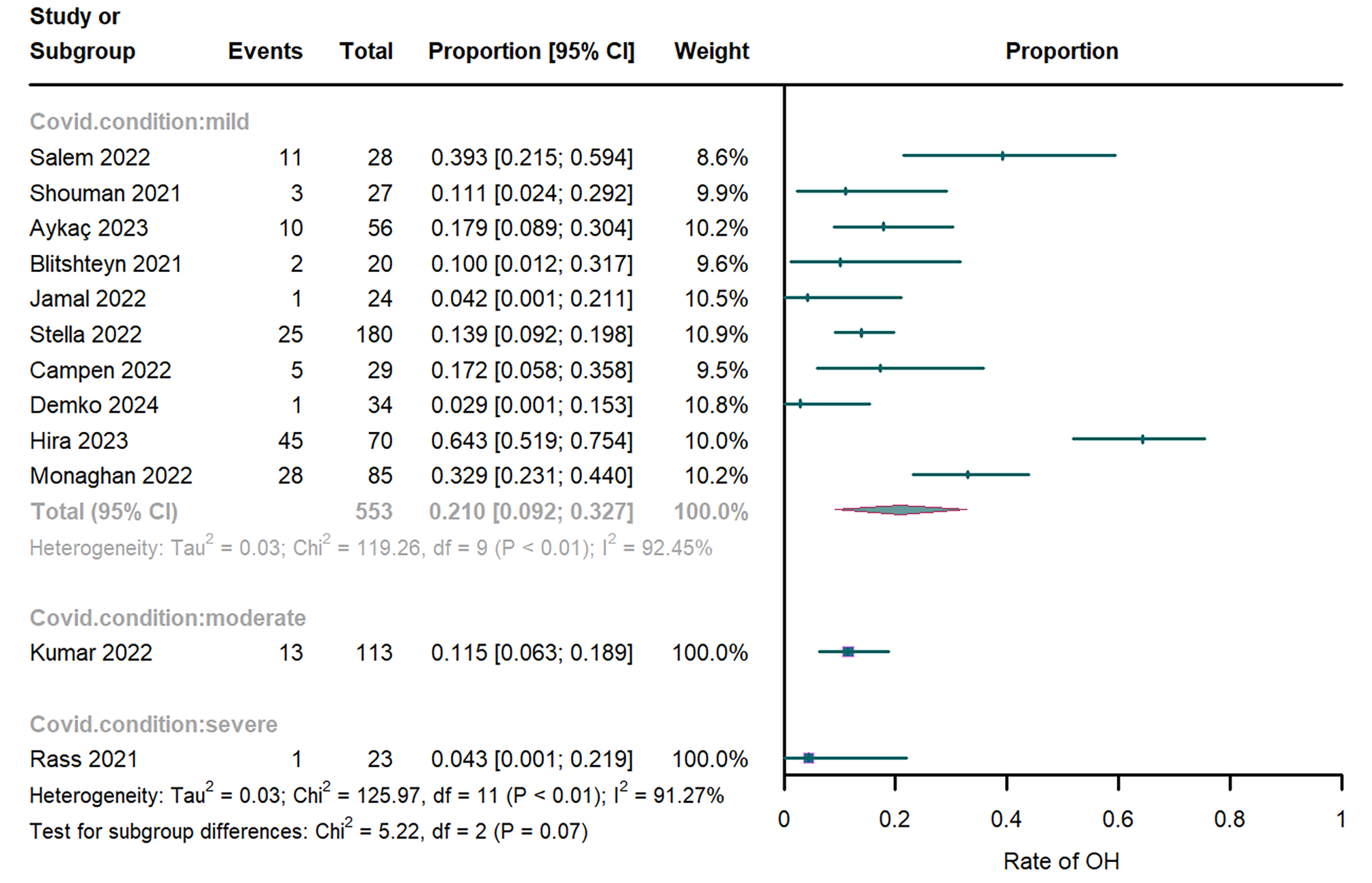


### **Supplementary Figure S5**: Forest plot of OH prevalence stratified by subtypes.

**
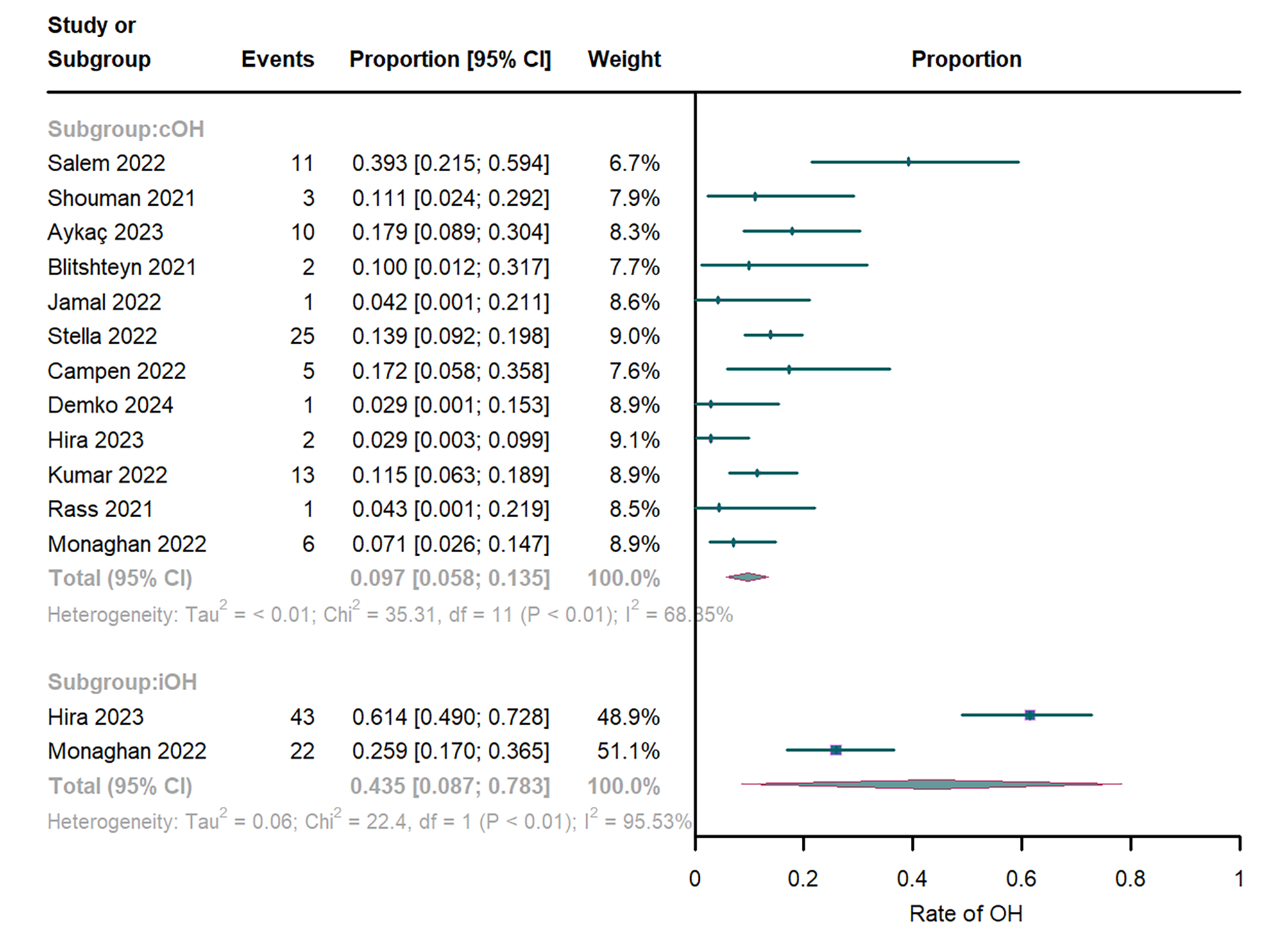
**

### **Supplementary Figure S6:** Sensitivity analyses of POTS prevalence.


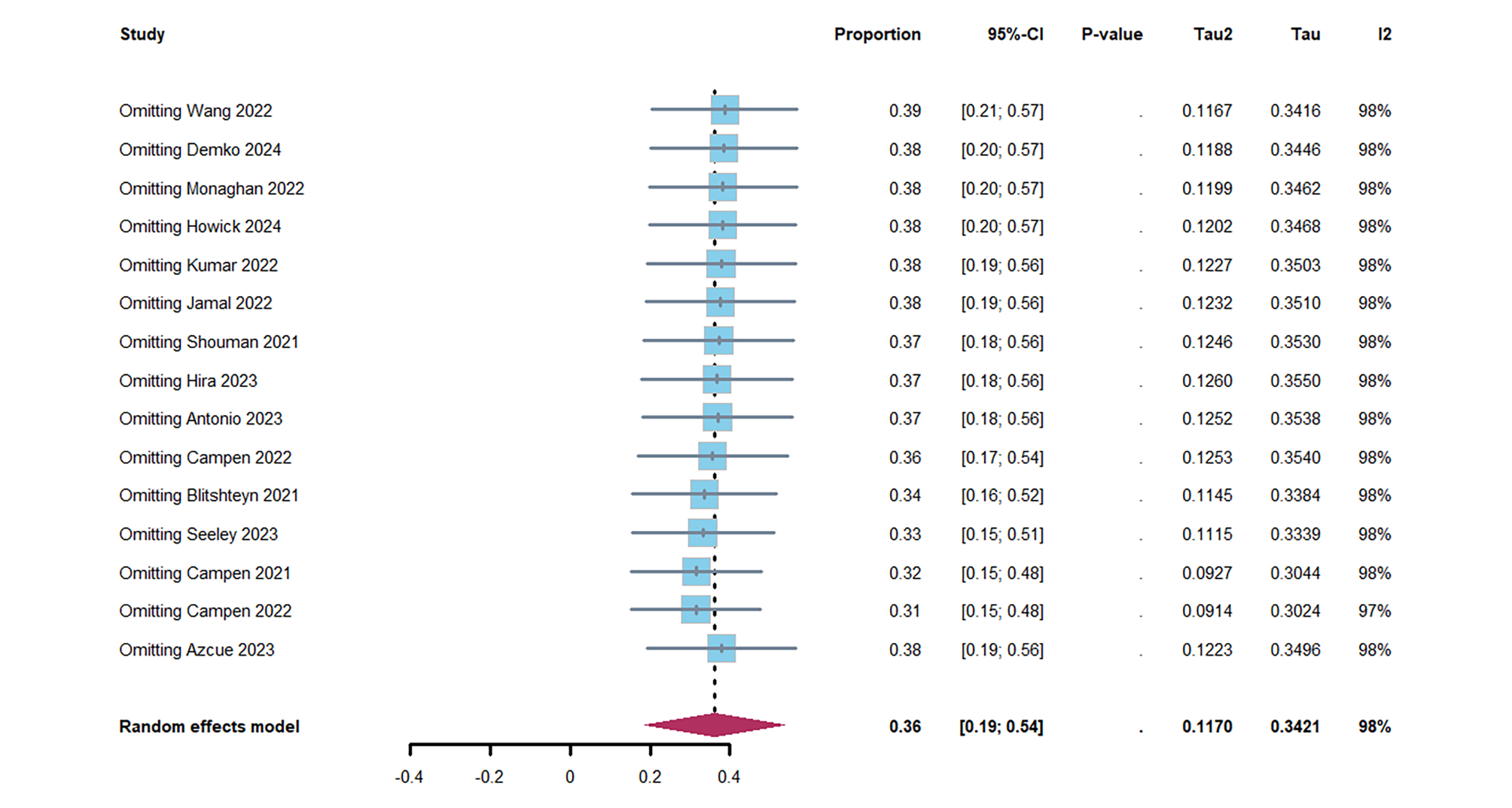


### **Supplementary Figure S7:** Sensitivity analyses of OH prevalence.

**
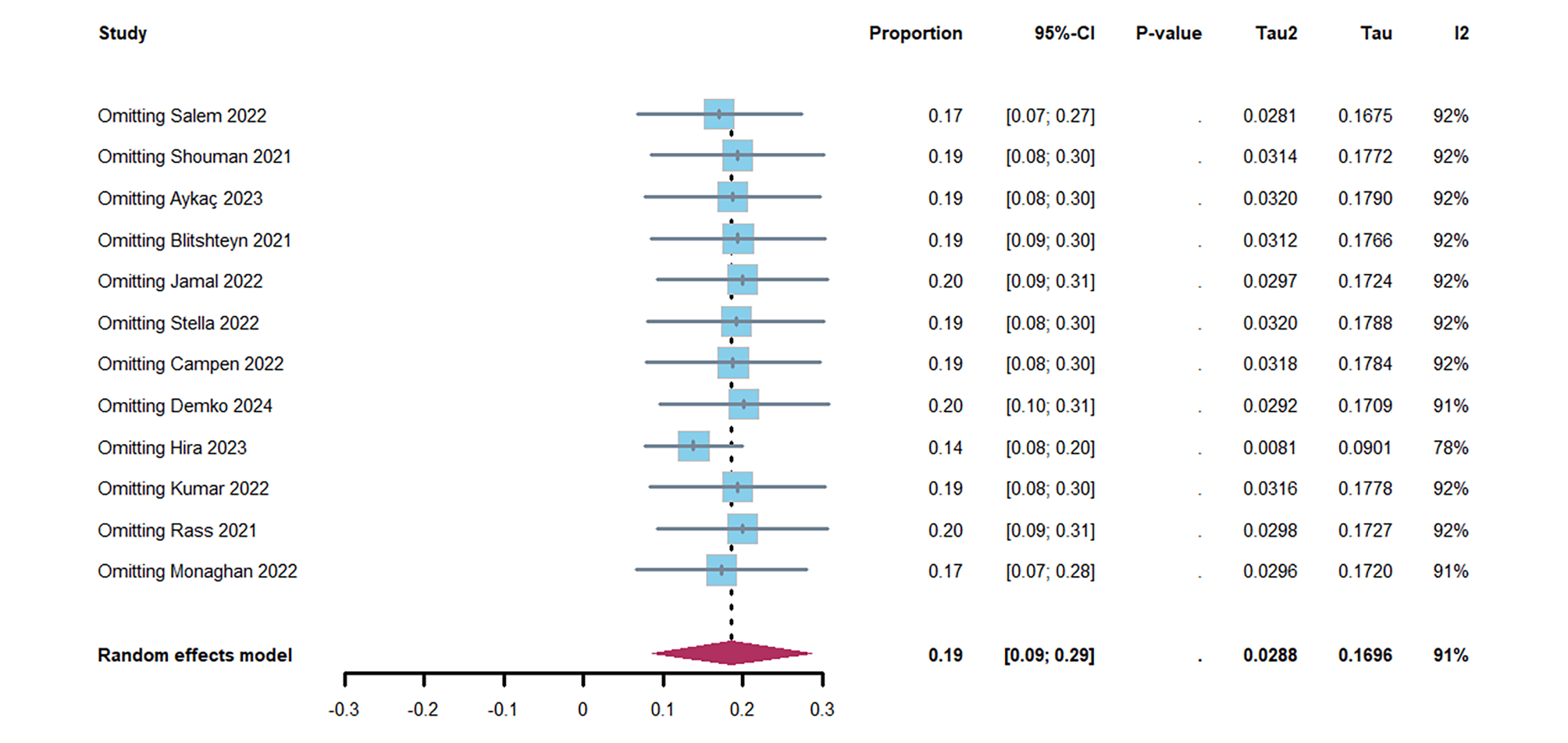
**

### **Supplementary Figure S8:** Bubble plots of POTS prevalence by mean age of participants.

**
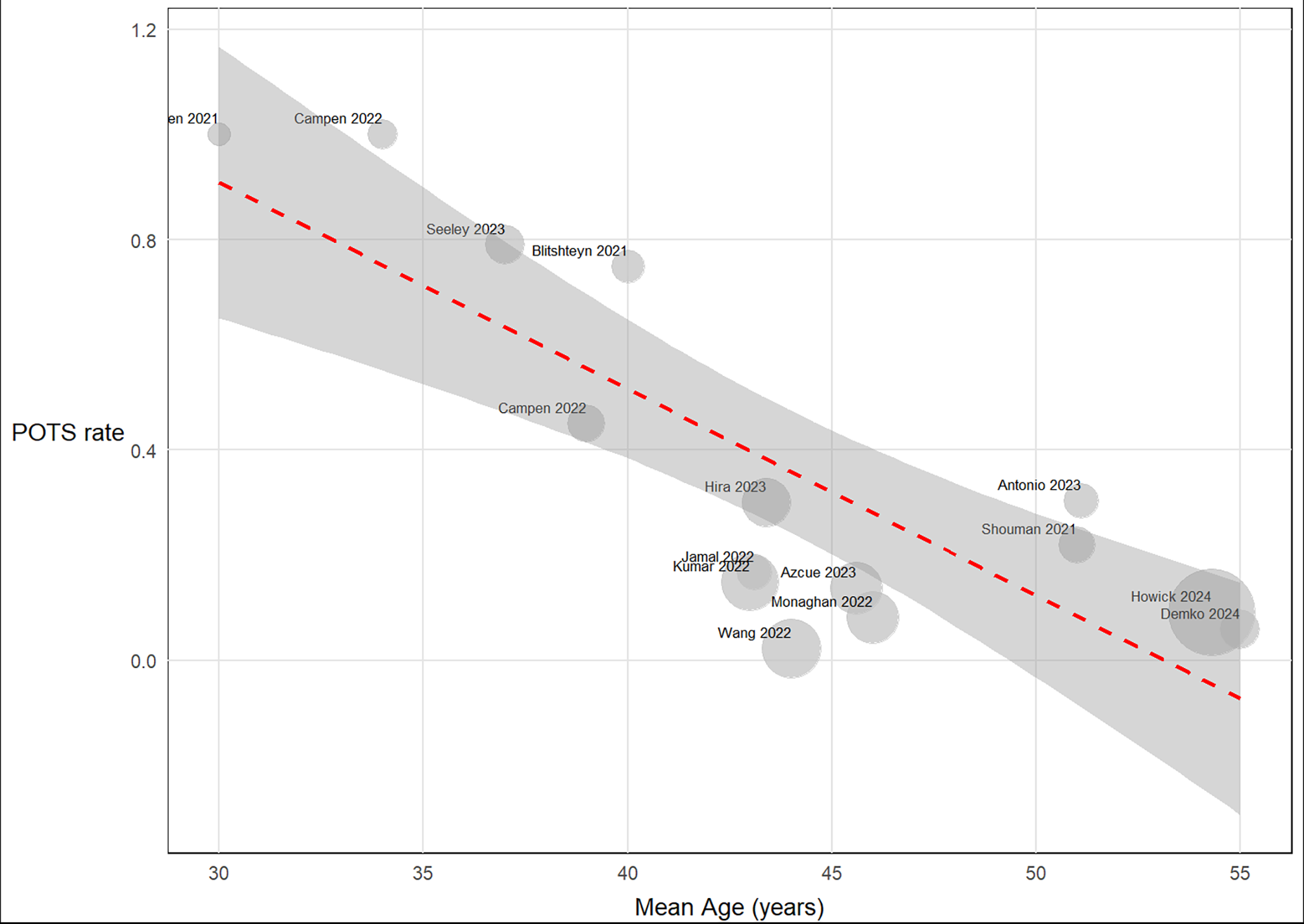
**

### **Supplementary Figure S9:** Bubble plots of OH prevalence by mean age of participants.

**
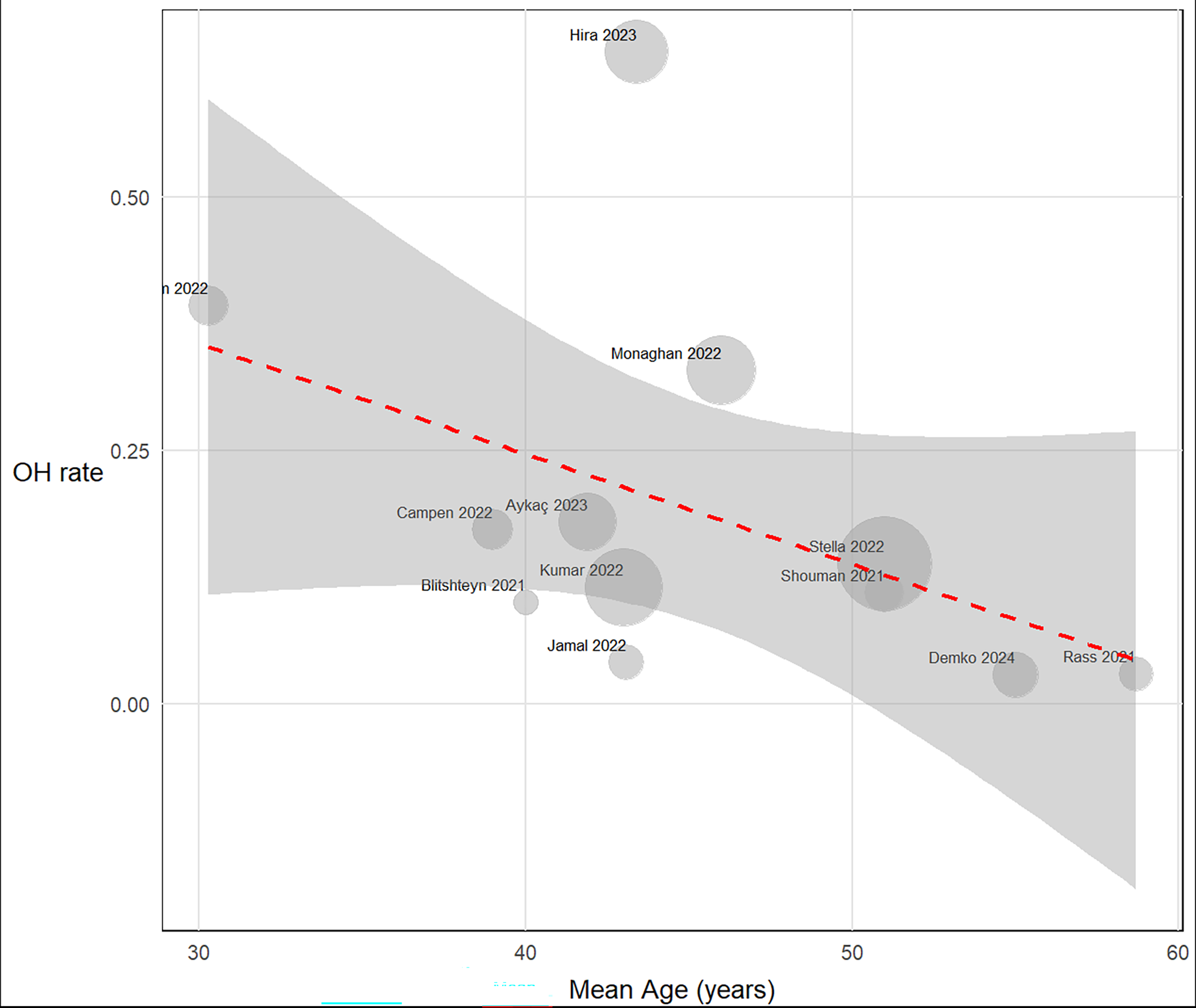
**

### **Supplementary Figure S10:** A: Egger's test for publication bias in POTS studies and B: The trim and fill funnel plot for POTS studies.

**
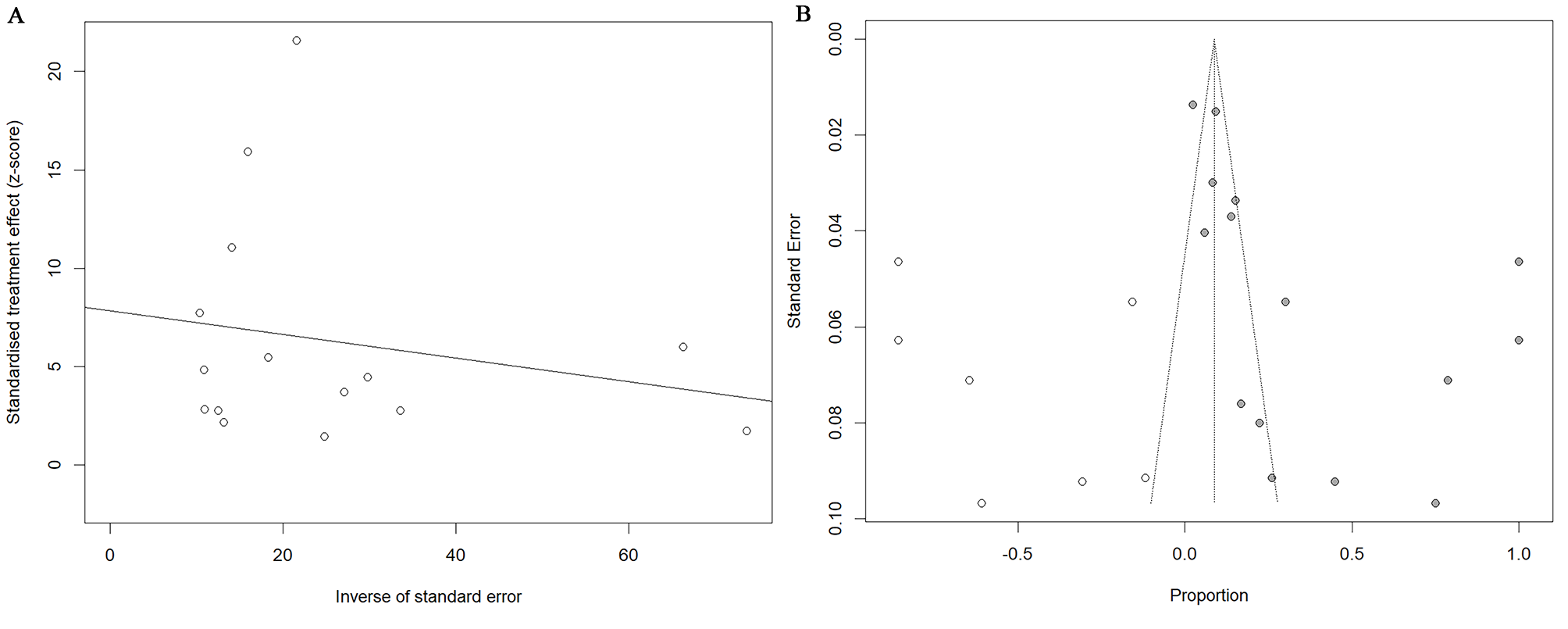
**
